# Supramolecular Polyphenol‐DNA Microparticles for In Vivo Adjuvant and Antigen Co‐Delivery and Immune Stimulation

**DOI:** 10.1002/anie.202214935

**Published:** 2023-02-10

**Authors:** Yijiao Qu, Robert De Rose, Chan‐Jin Kim, Jiajing Zhou, Zhixing Lin, Yi Ju, Sukhvir Kaur Bhangu, Christina Cortez‐Jugo, Francesca Cavalieri, Frank Caruso

**Affiliations:** ^1^ Department of Chemical Engineering The University of Melbourne Parkville Victoria 3010 Australia; ^2^ School of Science RMIT University Melbourne Victoria 3000 Australia; ^3^ Dipartimento di Scienze e Tecnologie Chimiche Universita' di Roma “Tor Vergata” Via della Ricerca Scientifica 1 00133 Rome Italy

**Keywords:** Drug Delivery, Hybrid Materials, Polyphenols, Self-Assembly, Vaccines

## Abstract

DNA‐based materials have attracted interest due to the tunable structure and encoded biological functionality of nucleic acids. A simple and general approach to synthesize DNA‐based materials with fine control over morphology and bioactivity is important to expand their applications. Here, we report the synthesis of DNA‐based particles via the supramolecular assembly of tannic acid (TA) and DNA. Uniform particles with different morphologies are obtained using a variety of DNA building blocks. The particles enable the co‐delivery of cytosine‐guanine adjuvant sequences and the antigen ovalbumin in model cells. Intramuscular injection of the particles in mice induces antigen‐specific antibody production and T cell responses with no apparent toxicity. Protein expression in cells is shown using capsules assembled from TA and plasmid DNA. This work highlights the potential of TA as a universal material for directing the supramolecular assembly of DNA into gene and vaccine delivery platforms.

## Graphical abstract

DNA‐based particles with various morphologies and functions are fabricated via the supramolecular assembly of tannic acid (TA) and DNA. The engineered particles enable in vivo co‐delivery of adjuvant/antigen to boost cytokine and antibody production.

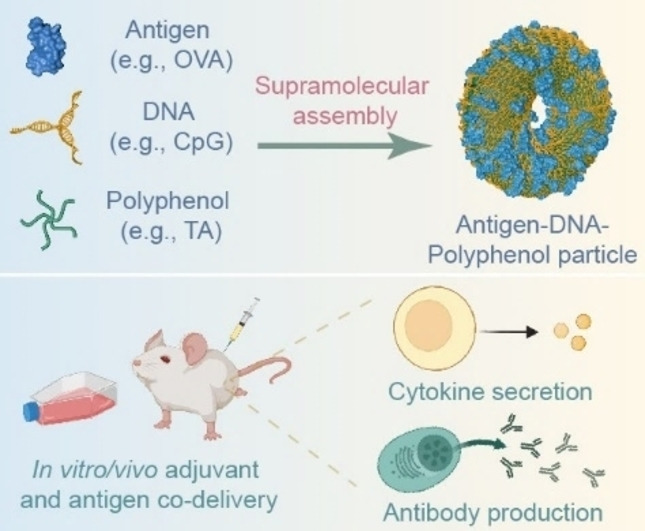

## Introduction

Nucleic acids are an important class of functional molecules with potential for a range of biomedical applications, including diagnostics and as active agents for the modulation of cell behavior at the genetic level, e.g., gene silencing or expression, immune modulation, and vaccines.^[1]^ For instance, the most promising vaccines currently used worldwide are those against coronavirus disease 2019 (COVID‐19), which use DNA and RNA in viral or non‐viral assemblies to express SARS‐CoV‐2 viral antigen.^[2]^ Naked or free DNA and RNA molecules cannot easily reach intracellular targets owing to their poor stability in the extracellular and intracellular environments (e.g., sensitivity to nucleases) and low intracellular uptake (which is inhibited by their negative charge and high molecular weight).^[3]^ Therefore, functional micro‐ and nanocarriers have been developed to serve as efficient nucleic acid delivery vehicles to protect the payload from degradation and improve cellular uptake.^[3]^ In particular, micrometer‐sized particles are readily taken up by macrophages and dendritic cells (DCs) (examples of TLR9 positive cells) and can potentially enable the efficient loading and co‐delivery of nucleic acid antigens and adjuvants.^[4]^ Unlike nanoparticles of <200 nm that typically drain to the lymph nodes after injection, micrometer‐sized particles are localized in the muscle, internalized by DCs, and ultimately transferred in the lymph nodes to induce an immune response.^[4]^ Biomolecules are ideal building blocks for the construction of advanced gene and protein delivery vehicles because of their excellent biocompatibility and biodegradability.^[5]^ In particular, polyphenols are abundant, cost‐effective naturally occurring compounds, with over 8000 unique molecules that have been employed in traditional medicine, tea and wine making, and other industries.^[6]^ Recently, there has been a growing interest in polyphenol‐based materials (e.g., particles, films, and hydrogels) owing to the universal adherent property of polyphenols and their potential biomedical applications.^[7]^ The catechol and galloyl moieties in polyphenols enable a range of interactions with diverse materials or substrates such as hydrogen bonding, metal coordination, and electrostatic interactions.^[6]^ Among the different polyphenols studied, tannic acid (TA) has been explored as a building block in the development of carriers for transporting various drugs, enzymes, and DNA.^[8]^ For example, Shin et al. have demonstrated the use of TA and DNA to form DNA hydrogels.^[9]^ Han et al.^[10]^ reported the preparation of TA‐DNA nanocomplexes via self‐assembly for gene release in cancer cells. However, to our knowledge, microparticle systems solely based on DNA and polyphenols has not been reported. Multifunctional particles based on DNA and polyphenols that are scalable and simple to synthesize could enable intracellular co‐delivery of antigens, adjuvants, and genes for a range of biomedical applications.

We recently demonstrated a template‐mediated assembly strategy to form pure DNA capsules comprised of unmethylated cytosine‐guanine (CpG) sequences as vaccine adjuvants.^[11]^ Although the DNA capsules exhibited high immunostimulatory activity, they required specifically designed DNA sequences as cross‐linkers for capsule stability and had limited capability in loading a wide range of nucleic acids or the co‐delivery of various types of cargos, e.g., proteins and peptides, which is challenging.^[12]^ Based on this premise, we hypothesize that supramolecular self‐assembly beyond conventional complementary base pairing could be leveraged for designing hybrid DNA particles to expand their applications.^[13]^ Accordingly, in the current study, we report a template‐mediated self‐assembly strategy to generate DNA‐TA particles stabilized by supramolecular interactions without the use of extraneous agents. Different morphologies (e.g., capsules and toroids) of DNA‐TA particles are prepared using nucleic acids with different molecular weights (e.g., 46 nt to 3 kbp) and structures (e.g., single‐stranded DNA (ssDNA), double‐stranded DNA (dsDNA), Y‐shaped double‐stranded DNA (yDNA), and plasmid DNA). The assembled DNA‐TA particles are highly resistant to serum nucleases. The versatility of this approach for loading DNA is demonstrated by co‐delivering CpG and a model antigen ovalbumin (OVA) for immunological modulation of RAW264.7 cells, as well as plasmid DNA for gene transfection. In addition, the efficacy of the DNA‐TA particles as a vaccine delivery system was demonstrated in vivo in a mouse model. Although lipid nanoparticles represent the state‐of‐the‐art for nucleic acid‐based vaccines, they also have some challenges, including stability and side effects that warrant the investigation of alternative systems for vaccination.[^14^, ^15^] This work shows the engineering of DNA‐TA particle assemblies as a potential versatile platform for vaccines and gene delivery.

## Results and Discussion

### Synthesis and Characterization of DNA‐TA Particles

**Figure 1:**
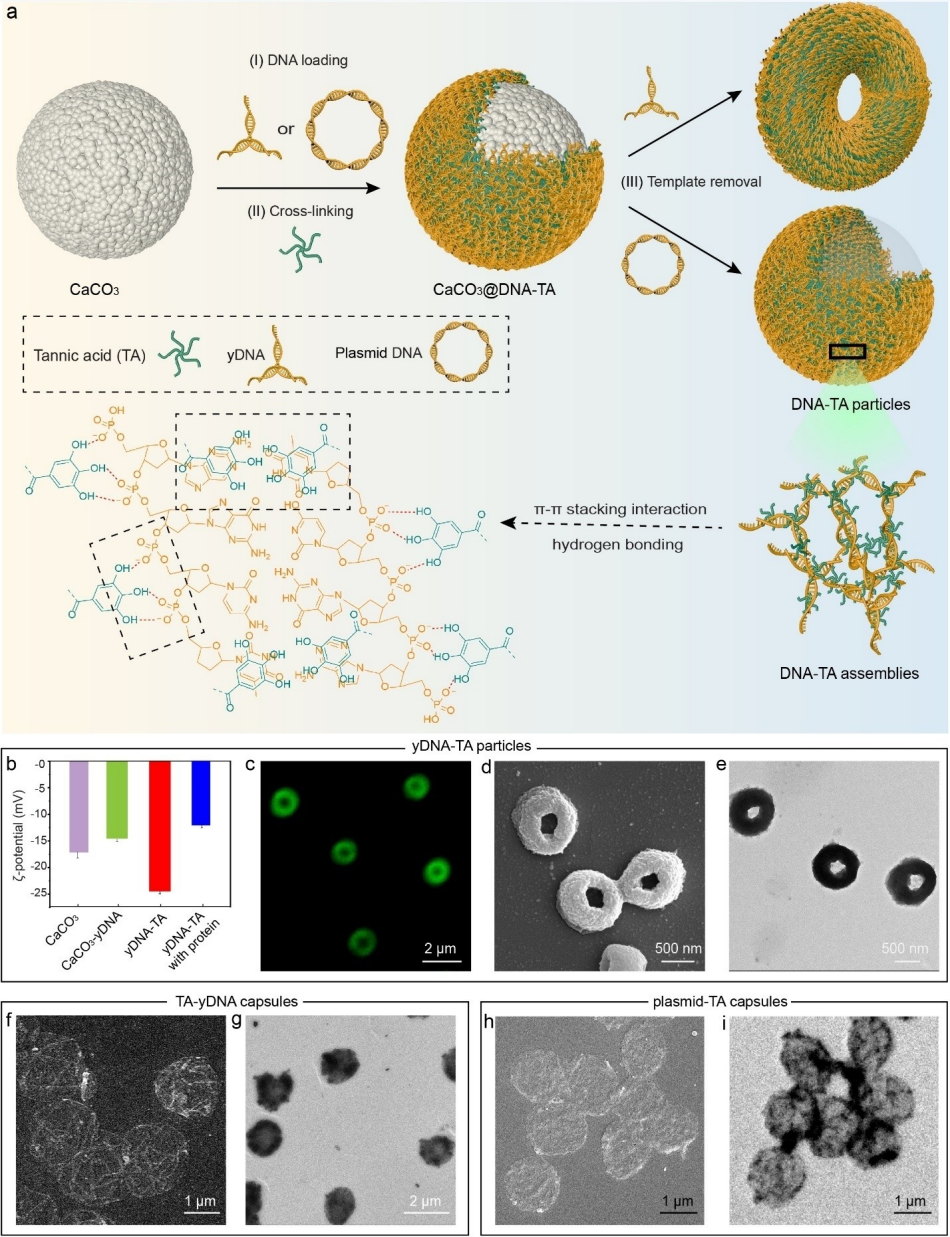
Preparation and characterization of DNA‐TA particles. a) Schematic representation of the assembly of DNA‐TA particles: (I) loading of CaCO_3_ particles with DNA (yDNA or plasmid DNA); (II) cross‐linking by TA; and (III) removal of CaCO_3_ template core to yield DNA‐TA particles. The possible interactions involved in the stabilization of the DNA‐TA assemblies are shown. b) ζ‐Potentials of the templates, yDNA‐loaded templates, yDNA‐TA particles (after template removal), and protein corona‐coated yDNA‐TA particles. c) CLSM, d) SEM, and e) TEM images of the yDNA‐TA particles. f) SEM and g) TEM images of the TA‐yDNA capsules. h) SEM and i) TEM images of the plasmid‐TA capsules.

Microparticles were prepared through template‐mediated supramolecular assembly of TA and yDNA. First, yDNA strands were prepared by hybridization of three complementary single strands (Table S1), which were then adsorbed onto the CaCO_3_ porous templates (2 μm in diameter). The particles were then mixed with an aqueous solution of TA to cross‐link the immobilized yDNA into a network, as shown in Figure 1a. Finally, the CaCO_3_ template was dissolved using ethylenediaminetetraacetic acid (EDTA) solution, yielding yDNA‐TA particles. The yDNA‐TA particles showed a negative zeta potential (−25 mV) in water (Figure 1b). This likely confers colloidal stability to the yDNA‐TA particles and prevents their aggregation in biological fluids containing serum proteins. By quantifying the amount of unbound yDNA in the supernatant, we estimate that each yDNA‐TA particle is composed of approximately 2.4 million yDNA units. Confocal laser scanning microscopy (CLSM) analysis of the yDNA‐TA particles (wherein yDNA was labeled with Alexa Fluor 488 dye (AF488)) showed that the particles were uniformly well dispersed in aqueous solution with a size of approximately 1.5±0.1 μm (Figure 1c). Scanning electron microscopy (SEM), transmission electron microscopy (TEM), and three‐dimensional CLSM images confirmed the well‐defined toroid‐like geometry of the yDNA‐TA particles (Figure 1d and e and Figure S1, respectively). The thickness of the yDNA‐TA particles was ≈270 nm, as measured by atomic force microscopy (AFM) (Figure S2). The interaction mechanism between DNA and TA was explored by dispersing the yDNA‐TA particles in a hydrogen bond breaking agent (e.g., urea), surfactants (e.g., Tween 20 and sodium dodecyl sulfate (SDS)), and organic solvents (e.g., tetrahydrofuran (THF) and dimethyl sulfoxide (DMSO)). As observed in Figure S3, the particles disassembled to different extents in 100 mM urea, SDS, Tween 20, DMSO, and THF, as confirmed by counting the number of intact particles by flow cytometry. The concentration of particles was approximately 1×10^7^ particles mL^−1^ in water. A drastic decrease in particle concentration (80–100 % reduction) was observed when yDNA‐TA particles were dispersed in various solvents (Figure S3). Considering the distinct behaviors of the yDNA‐TA particles in these media, we infer that both hydrogen bonding and π‐π stacking interactions are involved in stabilizing these particles.

The interaction between TA and yDNA within the porous CaCO_3_ templates results in thick and compact shells. We speculate that the toroidal morphology of the yDNA‐TA particles discussed above is due to shell collapse through the center of the particles. When EDTA is added to remove the templates, the pressure generated as a result of CO_2_ evolution inside the particles and the resistance to diffusion of gas through the shell might have caused shell breakage and collapse. Similar hollow polymeric structures with broken shells have been obtained through template‐assisted methods.^[16]^ The toroidal morphology of the yDNA‐TA particles was consistent across the range of CaCO_3_ template size examined during assembly. Both SEM and TEM images showed that the yDNA‐TA particles had a toroidal shape and shrank approximately 50 % after template dissolution (2.8 to 1.5 μm, 4 to 2 μm, and 7 to 3.2 μm) (Figure S4). Interestingly, microcapsules with thin shells (Figure 1f) were obtained when the reverse deposition sequence was used (i.e., TA followed by yDNA deposition; these particles are referred as TA‐yDNA capsules). This result indicates that morphological diversity can be achieved by simply switching the deposition order of the building blocks. The TA‐yDNA capsules with a thin shell and a diameter of approximately 1.5±0.2 μm are shown in Figure 1f and g. The thickness of the TA‐yDNA capsules was approximately 125 nm, as determined by AFM (Figure S5), which was significantly thinner than the yDNA‐TA particles discussed earlier (shell thickness of 270 nm). The initial adsorption of the highly adherent TA on the porous CaCO_3_ templates likely caused pore clogging, thereby inhibiting the effective subsequent deposition of yDNA, and thus leading to the formation of a thin and gas‐permeable shell. The formation of such thin shell likely facilitated the escape of CO_2_ produced during the dissolution of the template core, resulting in the formation of a capsule‐like morphology.

To further demonstrate the versatility (including morphological diversity potential) of the present method, a high molecular weight circular plasmid DNA (3 kbp) was used to synthesize plasmid‐TA capsules (plasmid loading followed by TA deposition). The plasmid‐TA capsules had thin shells and were approximately 1.5±0.1 μm in diameter, as determined by SEM and TEM (Figure 1h and i), with zeta potential of −25 mV in water. ssDNA and dsDNA were also successfully used to generate DNA‐TA capsules (Figure S6). Overall, these results indicate the general applicability of the present method to fabricate supramolecular assemblies of DNA with TA, two natural and biodegradable biomolecules, without resorting to other polymers. Uniform DNA‐TA particles with different morphologies were synthesized by using different types of DNA and molecular weights, including ssDNA, dsDNA, yDNA, and plasmid. This morphological diversity depends on the molecular weight (low or high) and structure (Y shape, circular plasmid, ssDNA, dsDNA) of DNA and its ability to efficiently interconnect TA molecules. The functionality of the different types of DNA combined with sequence specificity that can be encoded into DNA‐TA systems make them attractive for different applications such as immunotherapy and gene delivery.

#### Serum Stability and Cell Interaction of yDNA‐TA Particles

**Figure 2:**
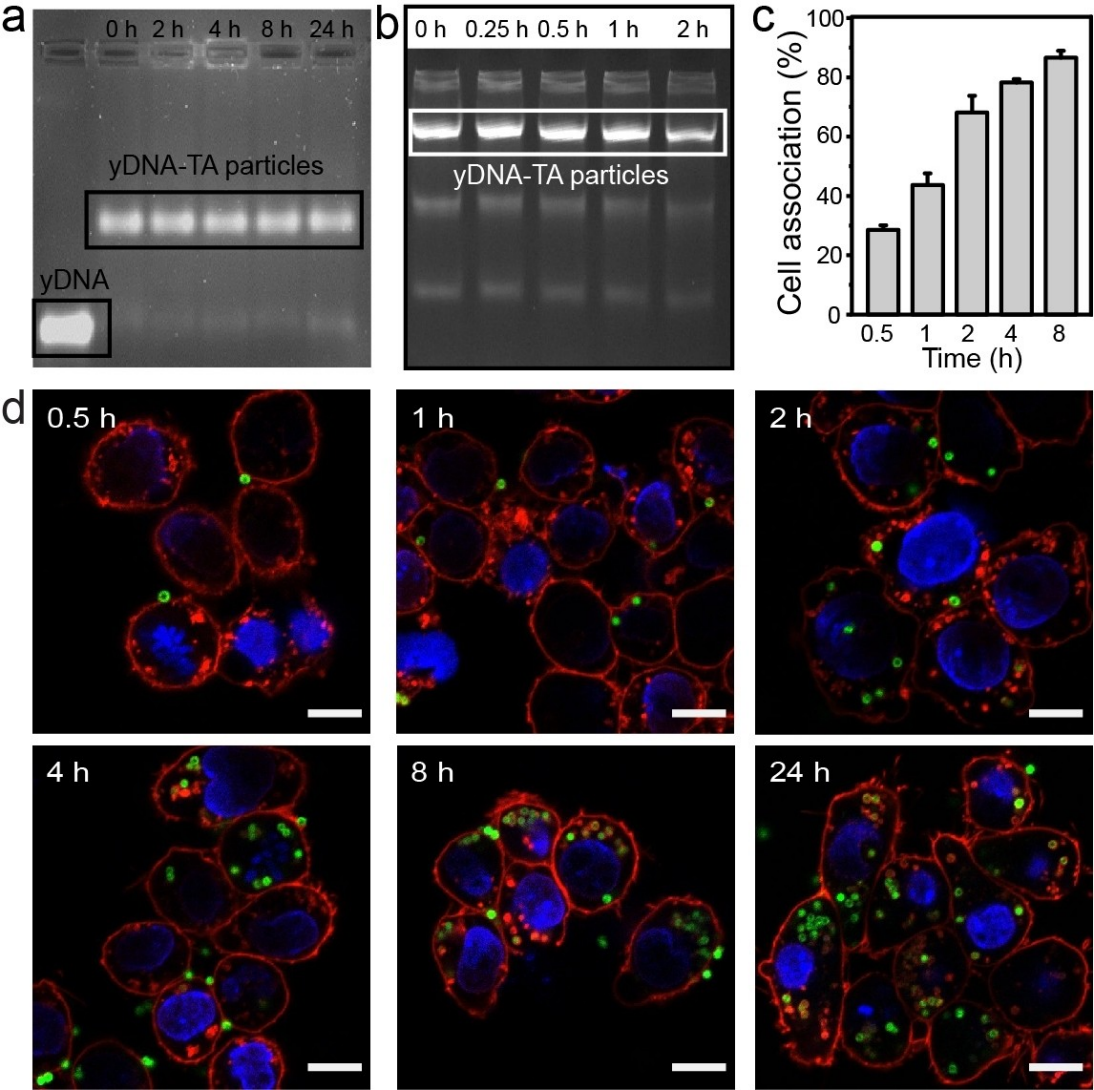
Stability and cellular interactions of the yDNA‐TA particles with RAW264.7 cells. a) PAGE analysis of the yDNA‐TA particles incubated in 10 % FBS at 37 °C for 2, 4, 8, and 24 h. b) Agarose gel electrophoresis analysis of the yDNA‐TA particles incubated in DNase (1 U mL^−1^) at 37 °C for 0.25, 0.5, 1, and 2 h. c) Time‐dependent association of the yDNA‐TA particles with RAW264.7 cells. d) CLSM images of cells after incubation with the yDNA‐TA particles for 0.5, 1, 2, 4, 8, and 24 h. Green, AF488‐labeled yDNA‐TA particles; blue, cell nuclei; red, cell membrane. Scale bars: 10 μm.

Carrier stability in the extracellular environment is a prerequisite for effective intracellular drug delivery. Hence, the stability and biodegradability of the yDNA‐TA particles was investigated. After incubation with the cell culture medium containing 10 % fetal bovine serum (FBS), the ζ‐potential of the yDNA‐TA particles changed from −25 to −13 mV owing to the adsorption of proteins from the serum to form a protein corona on the particles (Figure 1b, “yDNA‐TA with protein”). The adsorption of serum proteins was confirmed by sodium dodecyl sulphate polyacrylamide gel electrophoresis (SDS‐PAGE) (Figure S7).^[17]^ The yDNA‐TA particles maintained their toroidal structure after incubation with 10 % FBS medium for 24 h at 37 °C, as observed in the CLSM and SEM images in Figures S8 and S9, respectively. The stability of the yDNA‐TA particles following incubation with 10 % FBS at 37 °C at pH 4.5 and 7.4 was also confirmed by agarose gel electrophoresis and CLSM (Figure 2a and Figures S10 and S11). Minimal release (<10 %) of yDNA was observed possibly owing to the displacement of yDNA from the particles surface by serum proteins. To investigate the biodegradability of the yDNA‐TA particles, the latter were incubated with a high concentration of DNase (1 U mL^−1^). As observed from Figures 2b and S12, the degradation of yDNA embedded in the particles in the presence of DNase was relatively slow (20 % degradation after incubation for 24 h). These results confirm the stability of the yDNA‐TA particles, which may be attributed to their compact structure that reduces the accessibility of yDNA to nucleases. In the case of endo/lysosomal compartments, which are rich in nucleases, the yDNA‐TA particles could be partially degraded. The high stability and biodegradability of the yDNA‐TA particles suggest their potential use for intracellular drug delivery.

The cytotoxicity of the yDNA‐TA particles was examined by incubating the particles with RAW264.7 or HeLa cells for 24 h. As observed from Figures S13 and S14, the yDNA‐TA particles showed no detectable cytotoxicity after incubation for 24 h at the different particle‐to‐cell ratios (i.e., from 50 : 1 to 400 : 1) examined. The internalization efficiency of the yDNA‐TA particles (via cell association experiments) was assessed by flow cytometry using AF488‐labeled yDNA. As shown in Figure 2c, the association of the yDNA‐TA particles with RAW264.7 cells was time‐dependent and the percentage of positive cells (yDNA‐TA particles associated) reached 50 % after 1 h of incubation and 91 % after 8 h of incubation. The cellular internalization of the yDNA‐TA particles as a function of incubation time (0.5, 1, 2, 4, 8, 24, and 36 h) was monitored by CLSM (Figures 2d and S15). After incubation for 0.5 h, negligible internalization of the yDNA‐TA particles was observed. An increase in the cellular uptake of the yDNA‐TA particles was observed thereafter, which increased with prolonged incubation time to 36 h (Figure S15). For example, 3.3±0.4 yDNA‐TA particles per cell were observed after incubation for 24 h. Cellular uptake of the yDNA‐TA particles occurs through phagocytosis, macropinocytosis, and clathrin‐dependent endocytosis, as suggested by the studies performed using endocytic inhibitors (Figure S16). Collectively, these results indicate that the yDNA‐TA particles are non‐cytotoxic to RAW264.7 cells in vitro and can be internalized through a combination of endocytic pathways within 2 h of incubation, with the internalization of particles increasing with longer incubation times.

### Degradation and Intracellular Trafficking of yDNA‐TA Particles

**Figure 3:**
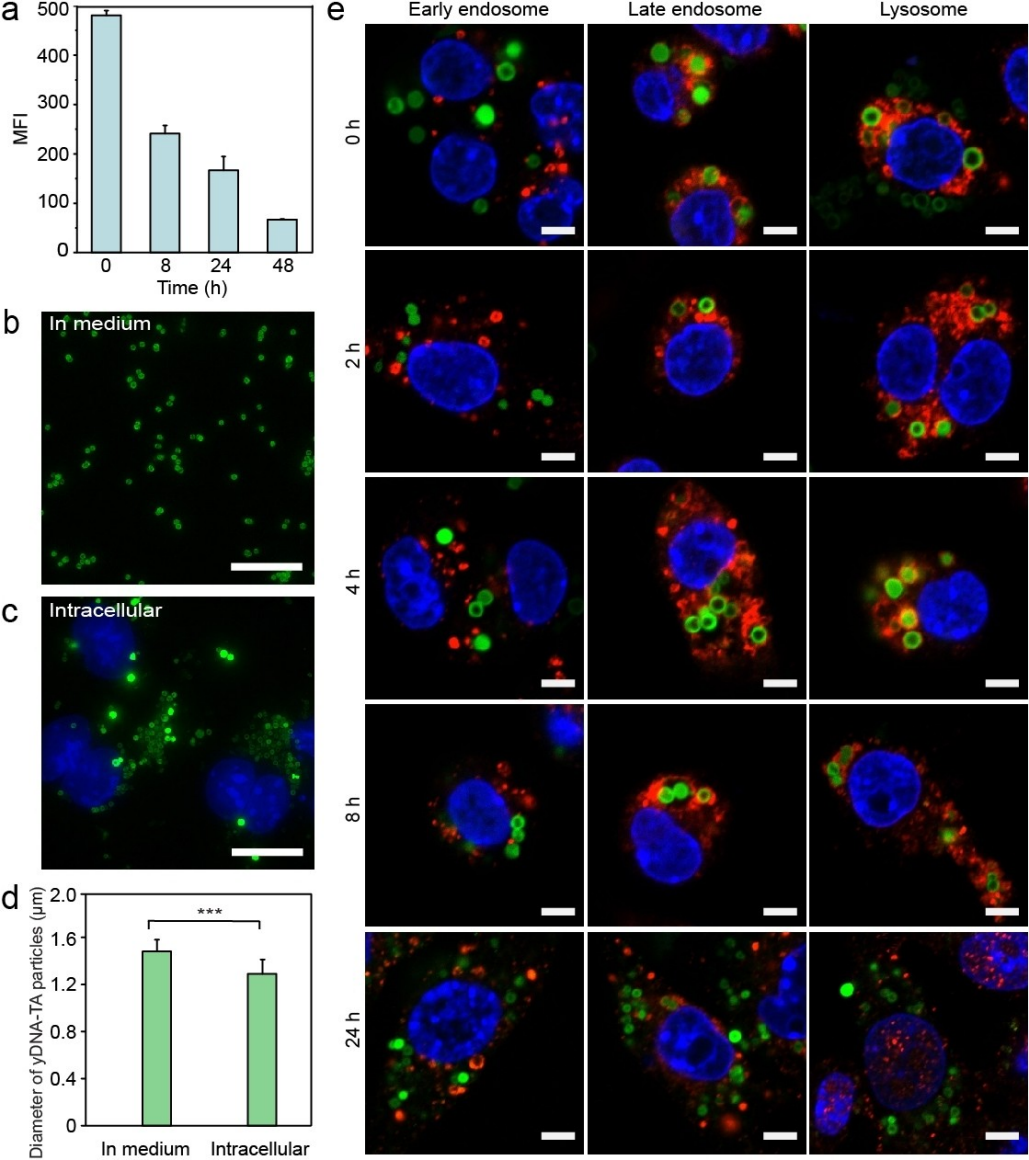
Intracellular degradation and trafficking of the yDNA‐TA particles in RAW264.7 cells. a) MFI of cells treated with the yDNA‐TA particles at varying incubation times of 0, 8, 24, and 48 h (following initial incubation for 2 h). SIM images of the yDNA‐TA particles (b) in the cell culture medium and (c) inside cells following incubation for 24 h. Scale bars: 20 μm. d) Diameter profiles of the yDNA‐TA particles in medium vs intracellular (data are shown as the mean±standard deviation, *n*>50). ***P<0.001. e) CLSM images showing the intracellular trafficking of the yDNA‐TA particles in RAW264.7 cells at varying incubation times of 0, 2, 4, 8, and 24 h (following initial incubation for 2 h). Compartments in cells (red) were stained with EEA1 monoclonal antibody (early endosomes), anti‐Rab7 monoclonal antibody (late endosomes), and anti‐LAMP1 monoclonal antibody (lysosomes). Green, AF488‐labeled yDNA‐TA particles; blue, nuclei; red, early endosomes or late endosomes and lysosomes. Scale bars: 5 μm.

The intracellular degradation of the yDNA‐TA particles was investigated by incubating RAW264.7 cells with AF488‐labeled yDNA‐TA particles. After 2 h of incubation, the non‐associated yDNA‐TA particles were washed out and cells were kept under incubation in fresh medium for 8, 24, and 48 h, respectively. The cells collected at different time points were fixed, and the mean fluorescence intensity (MFI) of the cells was measured by flow cytometry. As observed in Figure 3a, the MFI of the cells decreased with increasing incubation time, indicating that the yDNA‐TA particles were either degraded by the intracellular nuclease or eliminated through the process of exocytosis. To determine the size of the yDNA‐TA particles that were deployed in the cytosol with nanometer resolution, structured illumination microscopy (SIM) was used. After incubation with the cell culture medium, the yDNA‐TA particles did not show any change in size (1.5±0.1 μm) (Figure 3b). This confirms that the yDNA‐TA particles are stable in the extracellular environment. The diameter of the intracellular yDNA‐TA particles was smaller (i.e., 1.3±0.1 μm; Figure 3c and d). To directly visualize the colocalization of the yDNA‐TA particles with organelles, specific biomarkers of early endosome antigen 1 (EEA1), late endosome Ras‐related protein 7 (Rab7), and lysosomal‐associated membrane protein 1 (LAMP1) were used. Following incubation of cells with AF488‐labeled yDNA‐TA particles for 2 h, any unbound yDNA‐TA particles were washed out and the cells were kept under incubation in fresh medium for further 2, 4, 8, and 24 h. The cells were fixed at different time points, and endo/lysosomes were stained with the corresponding monoclonal antibodies. After a total incubation period of 10 h, the yDNA‐TA particles appeared to be surrounded by late endosomes and/or lysosomes but no appreciable colocalization with early endosomes was observed throughout the entire observation period (Figure 3e and Figure S17). The results suggest that after internalization, the yDNA‐TA particles rapidly translocated from early endosomes to late endosomes and lysosomes. Interestingly, after a prolonged incubation period (24 h), the yDNA‐TA particles did not show colocalization with the endocytic organelles and appeared smaller in size and less fluorescent. It is worth noting that the in vitro test (Figure S11) has shown the stability of the yDNA‐TA particles at pH 4.5 and 7.4, mimicking intracellular pH conditions. Overall, this study suggests that the yDNA‐TA particles may undergo partial degradation upon exposure to endo/lysosomal enzymes and subsequent translocation in the cytosol. A similar result was observed in HeLa cells—the yDNA‐TA particles appeared to escape the endo/lysosomes after 24 h (Figure S18). The limited pH buffering capacity of TA in the “pH change window” (pH 5–6.3) of the vesicles may facilitate endosomal escape by the “proton sponge effect” mechanism.^[18]^ However, the molecular basis of endosomal escape of the yDNA‐TA particle is not well understood and is being investigated. Collectively, the results indicate that the yDNA‐TA particles undergo erosion during endo/lysosomal trafficking and can potentially enable the release of nucleic acids (cargo) intracellularly.

### In Vitro Immunostimulatory Effect of CpG‐TA Particles

**Figure 4:**
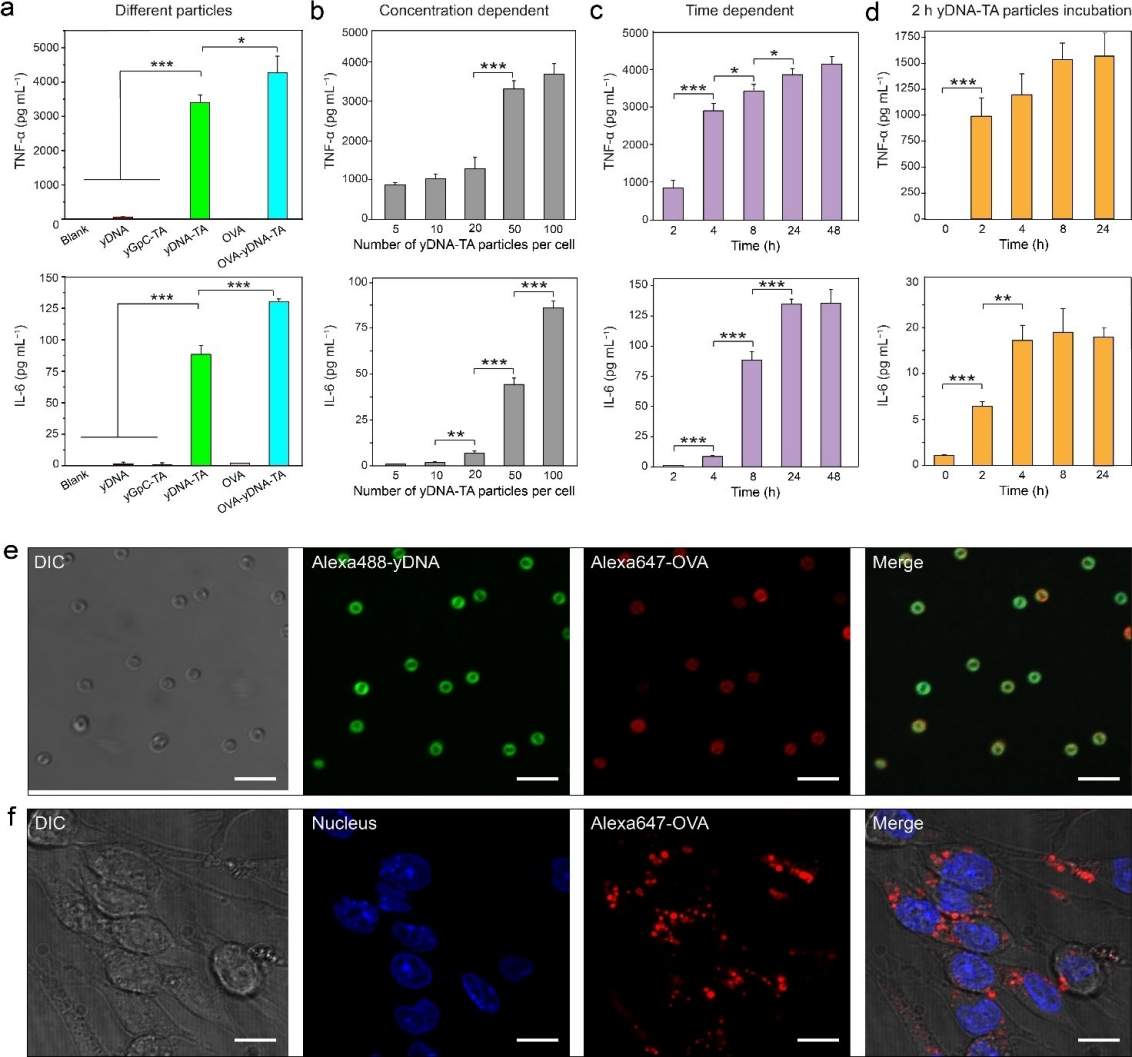
Cytokine release from RAW264.7 cells stimulated by the yDNA‐TA particles. a) ELISA measurements of TNF‐α and IL‐6 release stimulated by different DNA structures after incubation for 8 h (TNF‐α) or 24 h (IL‐6) with RAW264.7 cells (the concentration of the CpG motif in all samples was 120 nM; [OVA]=500 μg mL^−1^). b) TNF‐α and IL‐6 secretion from RAW264.7 cells incubated with the yDNA‐TA particles at varying particle‐to‐cell ratios for 8 h (TNF‐α) or 24 h (IL‐6). c) TNF‐α and IL‐6 secretion from RAW264.7 cells incubated with the yDNA‐TA particles ([CpG motifs]=120 nM, particle‐to‐cell ratio=100 : 1) for 2, 4, 8, 24 h and 48 h. d) TNF‐α and IL‐6 secretion from RAW264.7 cells incubated with the yDNA‐TA capsules ([CpG motifs]=120 nM, capsule‐to‐cell ratio=100 : 1) at varying incubation times of 0, 2, 4, 8, and 24 h (following initial incubation for 2 h). The results are expressed as the mean of cytokine concentration±standard deviation from three measurements. *t*‐test: *P<0.05, **P<0.01, and ***P<0.001. e) Differential interference contrast (DIC) and CLSM images of OVA‐yDNA‐TA particles; scale bars: 5 μm. f) DIC and CLSM images of cells after incubation with OVA‐yDNA‐TA particles for 24 h at a particle‐to‐cell ratio of 100 : 1; scale bars: 10 μm. Green, AF488‐labeled yDNA; red, AF647‐labeled OVA; yellow: merged yDNA and OVA; blue, nucleus.

The delivery of immunostimulatory unmethylated CpG motifs using the yDNA‐TA particles was then examined. Upon activation of the CpG motifs, immune cells (e.g., DCs and macrophages) secrete several cytokines, including tumor necrosis factor (TNF)‐α and interleukin (IL)‐6, which can in turn amplify the immune responses to vaccines and immunotherapies.^[19]^ CpG motifs are recognized by Toll‐like receptor 9 (TLR9), which is expressed and located in the endosomes of immune cells.^[20]^ To endow the yDNA‐TA particles with immunostimulatory capabilities, three CpG motifs were embedded in one yDNA building block. The immunostimulatory activities of the resulting yDNA‐TA particles were assessed by measuring the triggered secretions of cytokines (TNF‐α and IL‐6) in the culture medium of stimulated RAW264.7 cells using enzyme‐linked immunosorbent assay (ELISA). As observed in Figure 4a, the secretion of TNF‐α and IL‐6 upon incubation of RAW264.7 cells with yDNA was very low (TNF‐α: 60 pg mL^−1^ and IL‐6: 1 pg mL^−1^). In contrast, the yDNA‐TA particles induced the secretion of high levels (TNF‐α: 3400 pg mL^−1^ and IL‐6: 88 pg mL^−1^) of TNF‐α and IL‐6 in RAW264.7 cells. The difference in the immunostimulatory effect exerted by the yDNA‐TA particles compared with yDNA is attributed to the efficient cellular uptake of the yDNA‐TA particles (Figure 3e) and/or the susceptibility of the pristine yDNA to nuclease degradation. Furthermore, the yDNA‐TA particles stimulated the secretion of TNF‐α and IL‐6 in a concentration‐ and time‐dependent manner (Figure 4b and c). The dose and time dependency observed in our study is consistent with the hypothesis that a threshold of internalized capsules must be reached to observe maximum stimulatory effect.^[11]^

To investigate the TLR9 targeting specificity of the yDNA‐TA particles on immune activation, particles were prepared using yDNA that contained GpC dinucleotides (instead of CpG), denoted as yGpC‐TA particles, as a negative control. After incubation of an equivalent number of yGpC‐TA particles with RAW264.7 cells, negligible secretion of TNF‐α and IL‐6 was observed (Figure 4a). This result confirms that the immunostimulatory effect of the yDNA‐TA particles is mediated by specific interactions between CpG motifs and the TLR9. In addition, we examined whether the yDNA‐TA particles could exert prolonged immune stimulation effects in RAW264.7 cells after internalization. Cells were incubated with yDNA‐TA particles for 2 h, after which excess yDNA‐TA particles were washed out and cells were kept under incubation in fresh medium for another 2, 4, 8, and 24 h. The secretion of TNF‐α and IL‐6 from RAW264.7 cells at varying incubation times was then measured. As observed from Figure 4d, TNF‐α and IL‐6 were continuously produced at increasing levels within an incubation period of 8 h. Although the yDNA‐TA particles remain visually intact during intracellular trafficking, these findings indicate that the partial erosion of yDNA‐TA enabled cytokine production within 8 h of incubation. The negligible increase in cytokine production beyond 8 h is consistent with the observed translocation of the yDNA‐TA particles from the endo/lysosomes to the cytosol. Furthermore, DCs were incubated for 15 h with an equivalent number of yDNA‐TA and yGpC‐TA particles. The treated DCs were activated by yDNA‐TA, as observed in Figure S19, by upregulation of CD40, CD80, CD86, and MHC II receptors on the surface of the cells. In contrast, yGpC‐TA particles (containing the inactive sequence) did not induce increased expression of these receptors. Collectively, these results suggest that the yDNA‐TA particles can efficiently deliver bioactive CpG into RAW264.7 cells and DCs and induce a strong and sustained immune response.

For particulate vaccines, antigens and adjuvants are typically loaded in the same platform to induce a stronger immune response.^[21]^ To assess whether the combination of CpG motifs and an antigen within the yDNA‐TA particles can exert a synergetic effect in stimulating the immune system, OVA was employed as a model antigen in combination with the immune stimulatory effect of CpG.^[22]^ Owing to the protein binding activity of TA, OVA was post‐loaded in the yDNA‐TA particles (OVA‐yDNA‐TA particles) by deposition, followed by washing with water (Figure S20). Alexa Fluor 647 dye (AF647)‐labeled OVA was adsorbed onto the yDNA‐TA particles, as indicated by CLSM imaging (Figure 4e). The loaded amount of OVA on the yDNA‐TA particles (0.5 μg per 10^6^ particles) was calculated based on the standard curve calibrated with OVA solutions with concentrations ranging from 0.05 to 0.25 g L^−1^ (Figure S21). The stability of the OVA‐yDNA‐TA particles was compared with that of the yDNA‐TA particles in serum‐containing media (Figure S22). Interestingly, the OVA‐yDNA‐TA particles showed negligible disassembly up to 24 h incubation in serum compared to the yDNA‐TA particles. This is likely due to the enhanced structural stability conferred upon binding of OVA to yDNA‐TA. In addition, these results suggest that OVA is stably bound to the particles by non‐covalent stabilizing interactions with TA.^[8a]^ After incubation for 24 h with RAW264.7 cells, a strong fluorescence arising from the internalized OVA‐yDNA‐TA particles was observed (Figure 4f). This suggests that the OVA‐yDNA‐TA particles can be internalized in cells efficiently after 24 h. To verify the effect of OVA on the cellular uptake of particles, we analyzed the confocal microscopy images that show the internalization of yDNA‐TA and OVA‐yDNA‐TA particles in cells after 24 h incubation. We found that more OVA‐yDNA‐TA particles were internalized than yDNA‐TA particles (13±5 particles/cell vs. 8±3 particles/cell from analysis of >50 cells per treatment). The adsorption of OVA appears to improve the uptake of particles, which is likely an effect of reduced surface charge on the particles after protein adsorption (Figure 1b) and/or hydrophobic interactions between OVA‐yDNA‐TA particles and cell surfaces. The cytokine (TNF‐α and IL‐6) release induced by the OVA‐yDNA‐TA particles in RAW264.7 cells was higher when compared with that induced by the yDNA‐TA particles and free OVA (Figure 4a). Overall, this study suggests that upon partial degradation in the endosomes, the yDNA‐TA particles may release or expose CpG motifs that are recognized by TLR9 receptor and OVA fragments to ultimately trigger the immune response. These results show that the OVA‐yDNA‐TA particles provide a platform for the efficient co‐delivery of adjuvant and antigen.

To further demonstrate the potential of the DNA‐TA particles for delivering nucleic acids in the cytosol, a plasmid expressing enhanced green fluorescence protein (pEGFP, 3 kbp) was used as the DNA building block. Particles composed of pEGFP and TA (denoted as pEGFP‐TA capsules, Figure 1h and i) were incubated with HEK293T cells for 4 h, then the media was removed and replaced. Transfection efficiency was quantitatively determined by flow cytometry to assess the percentage of EGFP‐expressing cells 72 h post‐transfection. As shown in Figure S23, the transfection efficiency of the pEGFP‐lipofectamine complexes (a gold standard for transfection of plasmids into HEK293T cells) was 100 %, whereas that of the pEGFP‐TA capsules was 55 %. Notably, the pEGFP‐TA capsules showed a significantly higher transfection efficiency than free pEGFP (negative control). Consistent with the flow cytometry results, CLSM images of HEK293T cells transfected with the pEGFP‐TA capsules showed a strong fluorescence signal arising from EGFP expression. These results suggest that the pEGFP‐TA capsules, after trafficking through endocytic pathways, can disassemble in the cytosol to release bioactive pEGFP for expression of GFP. This also indicates that the pEGFP‐TA capsules remain intact extracellularly in the presence of 10 % FBS, although minimal release of pEGFP may be possible as observed with yDNA‐TA capsules (Figure 2a).

### In Vivo Immunogenicity of OVA‐yDNA‐TA Particles

**Figure 5:**
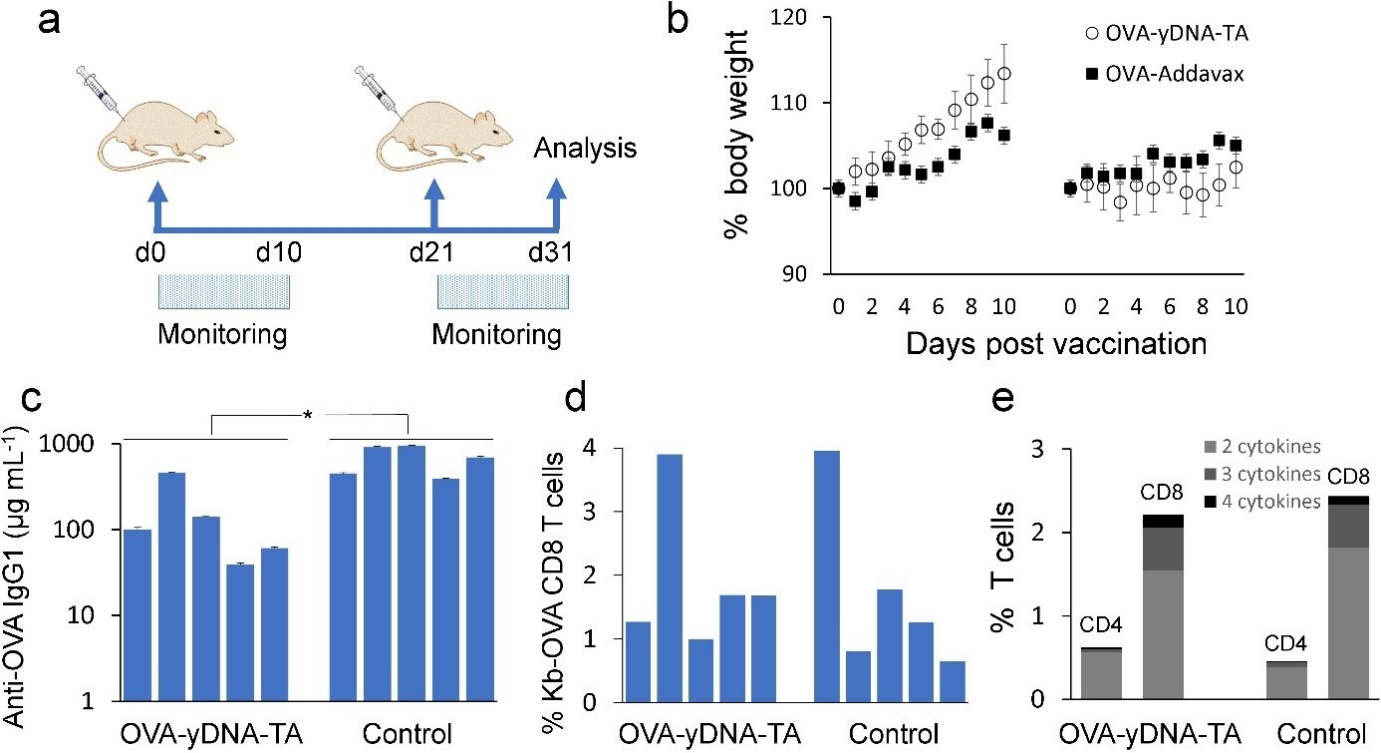
Immunogenicity of OVA‐yDNA‐TA microparticle vaccine. a) Schematic of vaccine regimen. Groups of 5 mice were vaccinated at Day 0 (d0) and Day 21 (d21). Blood and spleen were harvested at Day 31 (d31) for evaluation of immune response. b) Weights post vaccination. c) Antibody response in plasma. d) OVA‐specific CD8 T cell response (tetramer) and (e) polyfunctional T cell response in spleen. The particle vaccine was compared to soluble OVA formulated with AddaVax. Error bars in (b) and (c) represent standard deviations. * Indicates *p*=0.004.

Groups of 5 mice were vaccinated intramuscularly with OVA‐yDNA‐TA microparticles or positive control vaccine at Day 0 (d0) and Day 21 (d21),^[23]^ and immune responses were analyzed 10 days after the second vaccination (Figure 5a) when peak immune response is expected. The microparticle vaccine was well tolerated with no adverse events. Mice displayed increased weight after the first vaccination and stable weight after the second vaccination (Figure 5b). The immunogenicity of the microparticle vaccine was compared to OVA formulated with a strong adjuvant, AddaVax, a squalene‐based oil‐in‐water nanoemulsion 160 nm in diameter. The plasma antibody concentration induced by the OVA‐yDNA‐TA vaccine was 160 mg mL^−1^ compared to 679 mg mL^−1^ (*p*=0.004) for the control vaccine (Figure 5c). However, the T cell response induced by the OVA‐yDNA‐TA vaccine was equal to the control vaccine (Figure 5d). The percentage of OVA‐specific CD8 T cells induced by the OVA‐yDNA‐TA microparticles, as measured by Kb‐SIINFEKL tetramer, ranged from 0.99 to 3.90 % for the OVA‐yDNA‐TA vaccine and 0.65–3.95 % for the control vaccine (Figure 5d). Moreover, the quality of the T cell response, defined as the simultaneous expression of multiple effector molecules and a correlate for protection from disease,^[24]^ was comparable for both the particle and control vaccines. Polyfunctional expression of CD107a, a surrogate marker for degranulation, and the cytokines IFNγ, TNF, and IL‐2, was higher for CD8 T cells than for CD4 T cells (Figure 5e), suggesting that the vaccine is capable of inducing T cell memory suitable for protection from viral infection and neoantigens.

Initiation of immune responses requires antigen‐presenting cells to receive antigen plus an immunostimulatory molecule, as occurs with pathogens. Accordingly, OVA and CpG were packaged into the same particle to ensure activation of OVA‐presenting DCs to efficiently drive cognate T cells into a program of proliferation and differentiation. Moreover, the OVA‐yDNA‐TA microparticles carry a large amount of antigen and immunostimulatory molecules that can potentially improve vaccine efficiency. The initiation of immune responses may also have been assisted indirectly by local inflammation caused by macrophages. As observed in vitro, the incubation of microparticles with macrophages can induce secretion of the inflammatory cytokines TNF and IL‐6, that in turn will cause recruitment of other inflammatory cells and DCs.^[25]^ The size of the microparticle affects how antigens are exposed to the immune system and the type of immune response induced. Lymph node resident DCs have been shown to be crucial for priming cytotoxic T lymphocyte (CTL) responses but the size of the OVA‐yDNA‐TA microparticles precludes direct entry into lymph nodes.[^26^, ^27^] Instead, the OVA‐yDNA‐TA microparticles are likely internalized by DCs in the periphery by phagocytosis or micropinocytosis and migrate to the lymph node for engagement with cognate T cells.^[28]^ Our data shows that CTL and CD4 T cell responses are effectively induced by the OVA‐yDNA‐TA microparticles. It is also generally accepted that microparticles are more commonly associated with the induction of antibody responses.^[29]^ Yet, when compared to the control OVA vaccine formulated with a nanoparticle adjuvant, Addavax, the antibody response was lower. It is worth noting that the nanoformulation Addavax can drain directly to the lymph node for delivery of OVA to B cells, a requirement for antibody production.^[30]^ We speculate that the less efficient antibody response induced by OVA‐yDNA‐TA microparticles is due to the slow disassembly of microparticles in the muscle to release OVA that is ultimately drained to the lymph nodes. In summary, the OVA‐yDNA‐TA microparticles are non‐toxic and immunogenic, inducing both antibody production and T cell responses, which are prerequisites for an effective immune response.

## Conclusion

We have developed a simple and general method for synthesizing various DNA‐based microparticles by exploiting the non‐covalent interaction of polyphenols and DNA—the particles displayed diverse physicochemical properties and biological functions. Specifically, the particles exhibited high stability in serum likely due to their compact structure that can protect DNA from enzymatic degradation. The DNA‐TA particles were effectively internalized by macrophages and HEK293T cells, subsequently inducing cytokine production and protein expression, respectively, thereby highlighting their potential as a delivery platform. The applicability of the platform to a wide range of functional DNA types was demonstrated using CpG oligonucleotides, plasmid DNA, and a combination of oligonucleotides and protein cargos. In vivo studies revealed that the DNA‐TA particles are not toxic and act as a vaccine by inducing a good level of antibody production and T cells responses. The ease of preparation of the DNA‐TA particles combined with their tunable properties, stability in serum, and capacity for in vitro and in vivo intracellular co‐delivery of biomolecules offer a strategy for the loading of other nucleic acid‐based therapeutics, including messenger RNA and small‐interfering RNA for a range of applications.

Future in vivo studies will elucidate the fate of OVA‐yDNA‐TA capsules. As the engineered particles are made of degradable biomolecules, i.e., polyphenol, nucleic acids, and protein, we expect the OVA‐yDNA‐TA particles are ultimately degraded either in the extracellular environment (injection site) or in the intracellular endo‐lysosomal vesicles or cytosol of dendritic cells and other phagocytic cells and metabolized into degradation products (i.e., gallic acid, glucose, and amino acids) that can be used by the cells or eliminated by kidney filtration. In addition, our future work will explore size tunable next generation supramolecular particles that combine antigen‐expressing plasmid DNA or mRNA with CpG oligonucleotides and study the effect of size and cargo combinations on immune stimulation in vivo.

## Conflict of interest

The authors declare no conflict of interest.

1

## Supporting information

As a service to our authors and readers, this journal provides supporting information supplied by the authors. Such materials are peer reviewed and may be re‐organized for online delivery, but are not copy‐edited or typeset. Technical support issues arising from supporting information (other than missing files) should be addressed to the authors.

Supporting Information

## Data Availability

The data that support the findings of this study are available from the corresponding author upon reasonable request.
