# Supplementary material for: Supramolecular Polyphenol‐DNA Microparticles for In Vivo Adjuvant and Antigen Co‐Delivery and Immune Stimulation
Source: Angew Chem Int Ed Engl. 2023 Feb 10;62(12):e202214935. doi: 10.1002/anie.202214935 (PMC10946467; doi:10.1002/anie.202214935)
Supplement: Supplementary file 1 — Supporting Information [file ANIE-62-0-s001.pdf]

Angewandte Chemie
Eine Zeitschrift der Gesellschaft Deutscher Chemiker

Supporting Information

Supramolecular Polyphenol-DNA Microparticles for In Vivo Adjuvant and Antigen Co-Delivery and Immune Stimulation

Y. Qu, R. De Rose, C.-J. Kim, J. Zhou, Z. Lin, Y. Ju, S. K. Bhangu, C. Cortez-Jugo, F. Cavalieri, F. Caruso**

Table of Contents

Experimental Section S3
Results and Discussion S6
References S22
Author Contributions S22

Experimental Section

Materials. All oligonucleotides used in this study were synthesized and purified by Sangon Biotech Co., Ltd. (Shanghai, China). Tannic acid (TA), (3-(4,5-dimethylthiazol-2-yl)-2,5-diphenyltetrazolium bromide (MTT), agarose, dimethyl sulfoxide (DMSO), phosphate-buffered saline (PBS), $CaCl_2 \cdot 2H_2O$, Na_2CO_3, poly(sodium 4-styrenesulfonate) (Mw ~70000), Dulbecco's phosphate-buffered saline (DPBS), fetal bovine serum (FBS), bovine serum albumin (BSA), NaN_3, filipin from S. *filipinensis*, (N-ethyl-N-isopropyl)amiloride (EIPA), cytochalasin D, rat anti-lysosomal-associated membrane protein 1 (LAMP1) monoclonal antibody, ovalbumin (OVA), and ethylenediaminetetraacetic acid (EDTA) were purchased from Sigma-Aldrich (St. Louis, MO, USA). Pitstop 2 was purchased from Abcam (Cambridge, UK). Hoechst 33342, Dulbecco's modified Eagle's medium (DMEM), Alexa Fluor 594–wheat germ agglutinin (AF594-WGA), Lipofectamine RNAiMAX, and trypsin were obtained from Life Technologies. Rabbit anti-Rab7 monoclonal antibodies were purchased from Cell Signaling Technology (Danvers, MA, USA). Mouse anti-early endosome antigen 1 (EEA1) monoclonal antibody was purchased from BD Biosciences (San Diego, CA, USA). Enzyme-linked immunosorbent assay (ELISA) kits for tumor necrosis factor (TNF)-α and interleukin (IL)-6 were purchased from Invitrogen. ELISA kits for determination of anti-OVA IgG1 antibody were purchased from Sapphire Bioscience. Plasmid DNA (plasmid expressing enhanced green fluorescent protein (pEGFP), 3 kbp) was obtained from the Commonwealth Scientific and Industrial Research Organisation (CSIRO, Australia). MF59 adjuvant was purchased from Jomar Life Research, Iscove modified Dulbecco medium (IMDM) was purchased from Thermo Fisher Scientific, and Fc receptor blocking reagent was purchased from Miltenyi Biotec. CD45-BUV496 CD44-BB515, CD3-PerCP, CD4-R718, CD8a-BUV395, CD40-, CD80-, CD86-, and MHC II-fluorescently labeled antibodies were purchased from BD Biosciences. Brilliant Stain buffer was purchased from BD Biosciences. Permeabilizing Solution 2, IFNγ-BV711, TFN-APC, and IL-2-PE-Cy7 were purchased from BD Biosciences. All chemicals were used as received without further purification. Ultrapure water with a resistivity of greater than 18 MΩ cm was used in all experiments and obtained from a three-stage Millipore Milli-Q Plus 185 purification system.

Preparation of yDNA. The Y-shaped DNA (yDNA) building block containing cytosine–guanine (CpG) sequences was prepared by mixing three oligonucleotide strands yDNA-1, yDNA-2, and yDNA-3 (Table S1) in Tris-HCl buffer (10×10^{-3} M Tris-HCl, 1×10^{-3} M EDTA, pH 8.0, containing 150×10^{-3} M NaCl) and the final concentration of each strand was 20 µM. The mixture was heated at 95 °C for 5 min and slowly cooled to 4 °C at a rate of 1 °C min^{-1} to allow the strands to anneal to form yDNA strands. yDNA strands were then directly used without further purification. As a control, yGpC strands containing guanine–cytosine (GpC) sequences instead of CpG sequences were prepared by mixing three oligonucleotide strands (yGpC-1, yGpC-2, and yGpC-3, Table S1), as described above.

Template-Assisted Preparation of DNA–TA Particles. The DNA–TA particles were synthesized using $CaCO_3$ particles (2 µm) as templates (details of the synthesis can be found in previous study[1]). The $CaCO_3$ particles (1 mg) were suspended in 10 µL of Milli-Q water. Next, 20 µL of yDNA (20 µM) and 20 µL of 4-(2-hydroxyethyl)-1-piperazineethanesulfonic acid (HEPES) buffer (10 mM, pH 7.2, 500 mM NaCl, 50 mM $MgCl_2$) were added. The mixture was then incubated for 8 h under stirring. After incubation, TA (10 µL, 40 mg mL^{-1}) was added to the solution for 1 min. The coated $CaCO_3$ particles were washed with Milli-Q water three times to remove excess TA by centrifugation (2000 *g*, 2 min). The $CaCO_3$ template was dissolved by adding 100 µL of EDTA (100 mM, pH 4) to the pellet suspension. Following dissolution for 5 min at room temperature, the resulting yDNA–TA particles were washed with Milli-Q water three times by centrifugation (2000 *g*, 2 min).

Using 20 µL HEPES buffer and different DNA building blocks that is single-stranded DNA (ssDNA; 10 µL, 120 µM), double-stranded DNA (dsDNA; 30 µL, 20 µM), and plasmid (20 µL, 0.35 mg mL^{-1}), ssDNA–TA capsules, dsDNA–TA capsules, and plasmid–TA capsules were prepared, respectively, following the same protocol.

We determined that each DNA–TA particle is composed of approximately 2.4 million yDNA units and 4.166×10^{-8} µg plasmid (0.33 µg per cm^2) by quantifying the amount of unbound DNA in the supernatant and counting the number of particles via UV–vis spectroscopy and flow cytometry, respectively.

To prepare OVA–yDNA–TA particles, OVA (30 µL, 1 mg mL−1) was incubated with 20 µL of yDNA–TA particles (1×10^6 particles $µL^{-1}$) and Milli-Q water (150 µL, pH 4) at room temperature overnight under 500 rpm shaking, then washed with Milli-Q water (pH 4) three times by centrifugation (3000 g, 2 min) to remove unbound free OVA. The amount of adsorbed OVA on yDNA–TA particles was determined by calculating the amount of unbound OVA based on the absorbance peak of OVA at 220 nm. Then, the resulting OVA–yDNA–TA particles were redispersed in pH 4 Milli-Q water to get 400,000 particles $µL^{-1}$ containing 0.2 µg OVA $µL^{-1}$.

Characterization of Particles. An FEI Tecnai TF20 instrument at an operation voltage of 120 kV was used for transmission electron microscopy (TEM) analysis. Prior to TEM imaging, 5 µL of sample solution was deposited on formvar carbon-coated copper grids that were precleaned using plasma. A JPK NanoWizard II atomic force microscope was used to measure the thickness of the particles. Prior to atomic force microscopy (AFM) imaging, 10 µL of sample solution was dropped onto a clean glass substrate overnight and imaged with MikroMasch silicon cantilevers (NSC/CSC) in tapping mode. An FEI Quanta 200 field-emission scanning electron microscope operating at an accelerating voltage of 10 kV was used to image the particles. The dried samples were sputter-coated with gold prior to scanning electron microscopy (SEM) imaging. ssDNA–TA capsules, dsDNA–TA capsules, and plasmid–TA capsules were

characterized by the same method. The ζ-potentials of the particles were measured in Milli-Q water on a Zetasizer Nano-ZS instrument (Malvern Instrument, UK).

Sodium Dodecyl Sulfate–Polyacrylamide Gel Electrophoresis. To study the adsorption of serum proteins, a suspension (100 µL) of yDNA–TA particles was incubated in 1 mL DMEM containing 10% FBS for 1 h at 37 °C. The particles were then washed three times with PBS solution to obtain protein corona-coated particles. To strip the adsorbed proteins from the particles, NuPAGE LDS sample loading buffer was added to the particles and heated at 70 °C for 10 min. The particles were separated from the eluted proteins by centrifugation (2000 *g*, 2 min). After transferring the eluted proteins to a new tube, 50 mM dithiothreitol (DTT), a reducing agent, was added to cleave the disulfide bonds in the protein. DTT was incubated with the eluted proteins at 70 °C for 10 min. The samples were then loaded on a 10 % gel and ran at 200 V for 50 min in 1× Tris/Glycine/SDS running buffer.

Agarose Gel Electrophoresis. The stability of the yDNA–TA particles was analyzed by electrophoresis in a 1% agarose gel in 1 × Tris/borate/EDTA buffer (90 × 10^{-3} M Tris, 90 × 10^{-3} M boric acid, and 10 × 10^{-3} M EDTA, pH 8.0) at 80 V for 1 h. Then, the gel was stained with SYBR Gold following the manufacturer's protocol and imaged using a ChemiDoc XRS Imaging System (BioRad, USA). In the yDNA–TA particle degradation study, the products resulting from the incubation of the yDNA–TA particles with DNase (1 U mL^{-1}) for 0.25, 0.5, 1 and 2 h at 37 °C were loaded and electrophoresis was performed under the same conditions.

Cell Cultures. RAW264.7 cells, HeLa cells, and HEK293T cells were purchased from the American Type Culture Collection. RAW264.7 cells with a passage number of 18–36, HeLa cells with a passage number of 5–10, and HEK293T cells with a passage number of 23–35 were cultured in complete DMEM supplied with 10% FBS at 37 °C, 5% CO_2, and 95% humidity.

MTT Assays. RAW264.7 or HeLa cells were plated into 96-well culture plates at a density of 1 × 10^4 cells per well for 24 h. The yDNA–TA particles at different particle-to-cell ratios (10:1, 20:1, 30:1, 50:1, and 100:1) were added to cells and incubated for 24 h. After incubation, the cell culture medium was aspirated and MTT solution (150 µL, 0.5 mg mL^{-1}) was added to each well and incubated at 37 °C for 4 h. Then, 50 µL of DMSO was added to each well to dissolve the formazan crystals. A microplate reader (TECAN, InfiniteM200, Switzerland) was used to measure the absorbance of the supernatant at 570 nm.

Flow Cytometry Analysis. For the time course experiment, RAW264.7 cells were plated in a 24-well culture plate at a density of 1 × 10^5 cells per well for 24 h. The AF488-labeled yDNA–TA particles were then added at a particle-to-cell ratio of 100:1 and incubated with the cells for 2 h. The medium was then discarded and any unbound yDNA–TA particles were removed by washing the cells with PBS three times. Following a further 8, 24, and 48 h period of incubation, the fluorescence intensity of the cells was determined by flow cytometry.

Confocal Laser Scanning Microscopy (CLSM) Imaging. RAW264.7 cells were plated in Labtek 8-well chamber slides at 3 × 10^4 cells per well and incubated for 24 h. The medium was replaced with fresh culture media. The AF488-labeled yDNA–TA particles were added at a particle-to-cell ratio of 100: 1 for 0.5, 1, 2, 4, 8, 24, or 36 h of incubation. Then, cells were washed with PBS three times and fixed with 4% paraformaldehyde for 15 min. After gently washing with PBS three times, the cell membrane was stained with AF594–WGA (5 µg mL^{-1}) for 5 min and the cell nucleus was stained with Hoechst 33342 (1 µg mL^{-1}) for 10 min. The cells were then imaged with a Nikon A1R confocal microscope fitted with a 60 × 1.4 NA oil immersion objective.

Mechanism of Internalization of yDNA–TA particles. RAW264.7 cells were plated in a 24-well culture plate at a density of 5 × 10^4 cells per well for 24 h. Endocytosis inhibitors (pitstop 2, EIPA, filipin from *S. filipinensis*, NaN_3, and cytochalasin D) were added to the cells to achieve final concentrations of 5, 15, 12 µg mL^{-1}, 120 mM, and 25 µM, respectively. After 15 min incubation with the endocytosis inhibitors, the AF488-labeled yDNA–TA particles were added at a particle-to-cell ratio of 100:1 and incubated with the cells for 2 h. The culture medium was then removed and cells were washed twice with PBS and detached with trypsin. The cells were analyzed using a BD Accuri C6 flow cytometer.

Intracellular Trafficking Evaluated by CLSM. RAW264.7 cells were plated in Labtek 8-well chamber slides at 3 × 10^4 cells per well and incubated for 24 h. The medium was replaced with fresh standard media. The AF488-labeled yDNA–TA particles were added at a particle-to-cell ratio of 100:1 for 2 h. Then the cells were washed with PBS three times to remove unbound yDNA–TA particles and incubated further in fresh medium for 2, 4, or 8 h. Then, cells were fixed with 4% paraformaldehyde for 15 min and permeabilized with 0.1% Triton X-100 solution in PBS for 5 min, washing with 1% BSA in PBS three times during each step. Mouse anti-EEA1 monoclonal antibody, rabbit anti-Rab7 monoclonal antibody, or rat anti-LAMP1 monoclonal antibody (2.5 µg mL^{-1}) was added to the samples and incubated overnight. After washing with 1% BSA in PBS three times, cells were incubated with goat anti-mouse, goat anti-rabbit, or goat anti-rat AF647 conjugate antibody (2 µg mL^{-1}) for 1 h. Cells were gently washed with DPBS three times and incubated with Hoechst 33342 (1 µg mL^{-1}) for 10 min to stain the nucleus. The cells were imaged with a Nikon A1R confocal microscope equipped with a 60 × 1.4 NA oil immersion objective.

Cytokine Assays. RAW264.7 cells were plated into 96-well culture plates at a density of 5 × 10^4 cells per well and incubated overnight. Then, cells were incubated with yDNA, yDNA–TA particles, OVA, or OVA–yDNA–TA particles (CpG motifs equivalent 120 nM, OVA equivalent 500 µg mL^{-1}) in fresh medium. Cells were incubated at 37 °C for 8 h (for TNF-α analysis) or 24 h (for IL-6 analysis). The

levels of TNF-α and IL-6 in the supernatants were determined by ELISA using antibody pairs specific to these cytokines and protocols recommended by the manufacturer. For the concentration-dependent experiment, the yDNA–TA particles at different particle-to-cell ratios (10:1, 20:1, 30:1, 50:1, and 100:1) were added to cells and incubated at 37 °C for 8 h (for TNF-α analysis) or 24 h (for IL-6 analysis), and the cytokine level in the supernatants was measured. For the time-dependent experiment, the yDNA–TA particles at a particle-to-cell ratio of 100:1 were added to the cells and incubated at 37 °C for 2, 4, 8, and 24 h, and the cytokine level in the supernatants was measured. For the time course experiment, the yDNA–TA particles at a particle-to-cell ratio of 100:1 were added to the cells and incubated at 37 °C for 2, 4, 8, 24, and 48 h (following initial incubation for 2 h), and the cytokine level in the supernatants was measured.

In Vitro Activation of Dendritic Cells
DC1940 dendritic cells (5×10^5) were seeded into 48-well tissue culture plates and incubated with microparticles or diluent alone for 15 h. Cells were detached from the culture plate by incubating in 1.25 mM EDTA/PBS (1 mL) for 7 min, washed, and resuspended in PBS for antibody staining. Cells were incubated with Fc block (Becton Dickinson) for 5 min at room temperature to block nonspecific binding and subsequently in a cocktail of CD40-, CD80-, CD86-, and MHC II-fluorescently labeled antibodies for 1 h at room temperature. Cells were washed (and resuspended) in PBS/2 mM EDTA and acquired with a CytoFLEX LX cytometer (Beckman Coulter). Analysis was performed with FlowJoX, v10.8.1 to measure the relative expression of CD40, CD80, CD86, and MHC II receptors.

In Vitro Transfection by Flow Cytometry and Confocal Microscopy. HEK293T cells were plated in 24-well plates at a density of 1×10^5 per well overnight. pEGFP–TA capsules were then added to the cells (1 µg pEGFP per well) in serum-free DMEM. The lipofectamine–pEGPF complex was used as the positive control, whereas naked pEGFP was used as the negative control. Following incubation for 18 h, the serum-free media was replaced with fresh medium. After further incubation for 30 h, the transfected cells were harvested, and washed with PBS once. The gene transfection efficiency was evaluated by flow cytometry by gating for the percentage of cells expressing EGFP. For confocal microscopy analysis of transfection, HEK293T cells were plated in Labtek 8-well chamber slides at 2×10^4 cells per well and incubated overnight. pEGFP–TA capsules, lipofectamine-pEGFP, or naked pEGFP (1 µg pEGFP per well) were incubated with the cells as described above for flow cytometry. The transfected cells were imaged with a Nikon A1R confocal microscope fitted with a 60 × 1.4 NA oil immersion objective.

Immunization of Mice. Vaccines were formulated using endotoxin-free OVA (Ovalbumin EndoFit, InvivoGen). Particles were prepared using endotoxin-free water using pyrogen-free plasticware. Groups of 5 female C57BL/6 mice, 6–8 weeks old, were injected bilaterally with the vaccine (50 µL) into the thigh muscles. Mice received either OVA–yDNA–TA microparticles containing 10 µg of OVA or 10 µg soluble OVA formulated with MF59 adjuvant at Day 0 and Day 21. At Day 31, mice were euthanized, and blood and spleen were harvested for immunological assays. All research involving animals was approved by the University of Melbourne Animal Ethics Committee (project ID 22241) and conducted in accordance with the University's Animal Care and Use guidelines.

Tissue Processing. Blood was harvested by cardiac puncture into 1 mL lithium heparin BD microtainer tubes (BD Biosciences), centrifuged at 2500 *g* for 15 min and the plasma was stored at −80 °C. All plasma samples were tested simultaneously. Spleens were harvested, trimmed of fat and connective tissue, and placed into cold IMDM medium + 10% FBS. Splenocytes were extracted by cutting the spleen into 2 pieces with scissors, placed onto a prewetted 100 µm cell strainer (ProSciTech) sitting in a 50 mL Falcon tube and the tissue gently pressed and ground against the filter with the barrel of a 10 mL syringe using ample medium to keep the tissue moist. PBS was added to the tube and cells were pelleted at 400 *g* for 4 min. The cell pellet was resuspended in 1 mL ACK (ammonium-chloride-potassium) lysing buffer and incubated for 1 min to lyse erythrocytes, then washed and centrifuged again in PBS. The splenocytes were resuspended in IMDM medium + 10% FBS, counted by hemocytometer, and the concentration was adjusted to 2×10^6 cells mL^{-1} for T cell assays.

Immunological Assays—ELISA. The concentration of anti-OVA IgG1 antibody in plasma 10 days after the second vaccination was measured with a commercial kit (Sapphire Bioscience, Cat# 500830) according to manufacturer's instructions. Briefly, plasma (100 µL) was added to OVA-coated plates and incubated for 2 h at room temperature, washed 4 times and replaced with goat anti-mouse horseradish peroxidase (100 µL) for 1 h at room temperature. After a final wash, the TMB substrate (100 µL) was added for color development for 30 min followed by HCl (100 µL) to stop the reaction. The absorbance was measured at 450 nm. Triplicate samples were measured and the concentration (mg mL^{-1}) was extrapolated from the standard curve. An *F*-test was performed to confirm equality of variances for antibody concentrations between the two vaccine groups, followed by a student *t*-test to compare means for significant differences.

Tetramer. Total OVA-specific CD8 T cells were enumerated by tetramer staining. Viakrome-808 (2 µL; Beckman Coulter) was added to 10^6 splenocytes in PBS and incubated for 20 min at room temperature to identify viable cells. Cells were washed in PBS at 400 *g* for 4 min, resuspended in staining buffer (PBS + 2% FBS + 2 mM EDTA) and chilled on ice prior to staining for 1 h with the H-2Kb-SIINFEKL tetramer labeled with PE, a gift from Professor Andrew Brooks, Department of Microbiology and Immunology, University of Melbourne. Cells were washed and incubated on ice in staining buffer (50 µL) containing Fc receptor blocking reagent (10 µL), followed by surface labeling for 1 h on ice with CD45-BUV496 (1:400 dilution), CD44-BB515 (1:400), CD3-PerCP (1:200), CD4-R718 (1:200), and CD8a-BUV395 (1:200). Brilliant Stain buffer was added to the antibody staining cocktail according to manufacturer's instructions.

Antibody-labeled cells were washed in staining buffer and resuspended in PBS + 2 mM EDTA for acquisition on a CytoFLEX LX cytometer (Beckman Coulter). FlowJoX software, v10.8.1, was used to measure the percentage of OVA-specific CD8 T cells.

Intracellular Cytokine Staining. Functional OVA-specific CD4 and CD8 T cell immune responses were analyzed by re-stimulating expression of IFNγ, TNF, CD107a, and IL-2. Splenocytes (2 × 10^6) in IMDM media + 10% FBS (100 ∝L) were incubated with OVA peptides 257-264 and 323-339 (10 μg mL^{-1}; Mimotopes) for 5 h at 37 °C/5% CO_2 or PBS alone for background subtraction. The media was supplemented with brefeldin A (0.2 μL; GolgiPlug, BD Biosciences) + monensin (0.133 μL; BD Biosciences) and CD107a-BV421 antibody (0.2 μL; BD Biosciences). Positive controls were stimulated with PMA + Ionomycin (0.4 μL, leukocyte activation cocktail, BD Biosciences). Following activation, cells were washed (400 *g* for 4 min) and resuspended in Viakrome-808 viability dye (1 μL) in PBS for 20 min at room temperature. Cells were washed and incubated on ice in staining buffer (50 μL) containing Fc receptor blocking reagent (10 μL) followed by surface labeling for 1 h on ice with CD45-BUV496 (1:400 dilution), CD44-BB515 (1:400), CD3-PerCP (1:200), CD4-R718 (1:200), and CD8a-BUV395 (1:200) and Brilliant Stain buffer. Cells were washed, fixed with 4% paraformaldehyde for 20 min at 4 °C, washed again and stored overnight in PBS + 2% FBS at 4 °C. Cells were pelleted at 1500 *g* for 5 min (and all subsequent washes) and permeabilized in Permeabilizing Solution 2 (100 μL) for 20 min on ice, then washed again. Antibodies for intracellular staining, IFNγ-BV711, TFN-APC, and IL-2-PE-Cy7, were prepared in Permeabilizing Solution 2 and added to cells for 30 min on ice, washed and resuspended in PBS + 2 mM EDTA. Cells were acquired with a CytoFLEX LX cytometer and analyzed with FlowJoX, v10.8.1 to determine the percentage of OVA-specific CD4 and CD8 T cells expressing two or more functional mark.

Minimum Information Reporting in Bio-Nano Experimental Literature (MIRIBEL). To improve reproducibility, reporting, and re-analysis, this study conforms to the MIRIBEL standard,[2] and a companion checklist is provided herein.

Results and Discussion

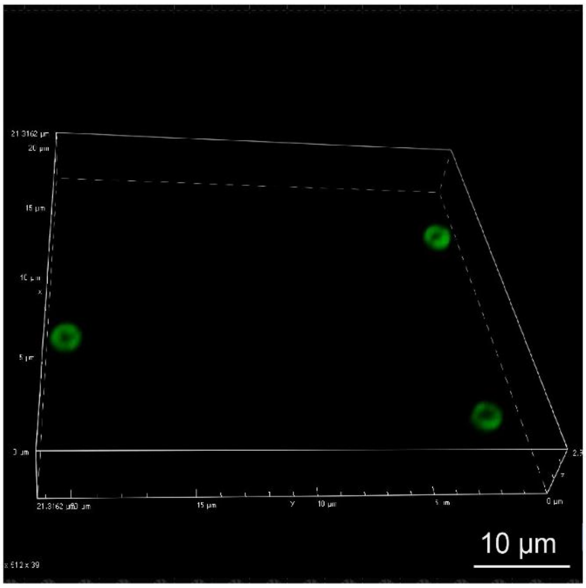

Figure S1. Three-dimensional CLSM image of the yDNA–TA particles.

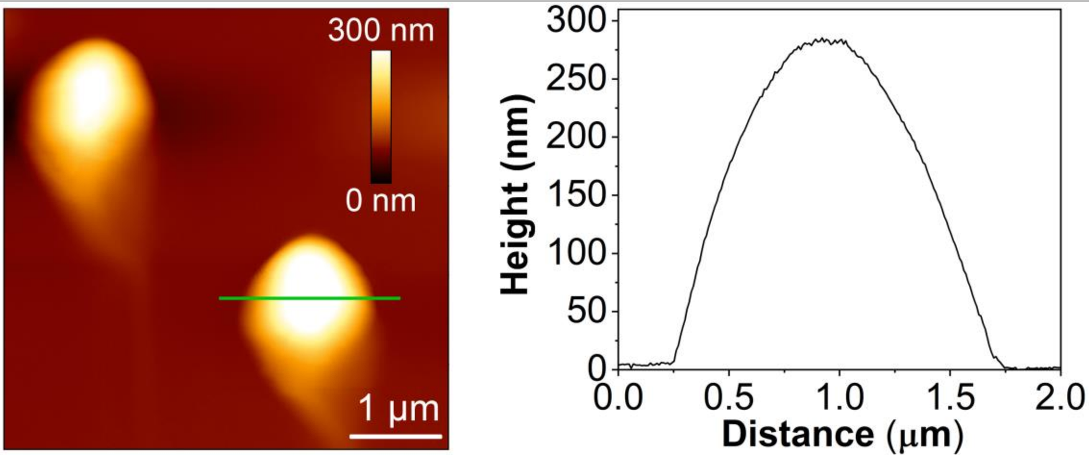

Figure S2. AFM image of the yDNA–TA particles. Height versus distance profile of a yDNA–TA particle plotted along the green line in the AFM image.

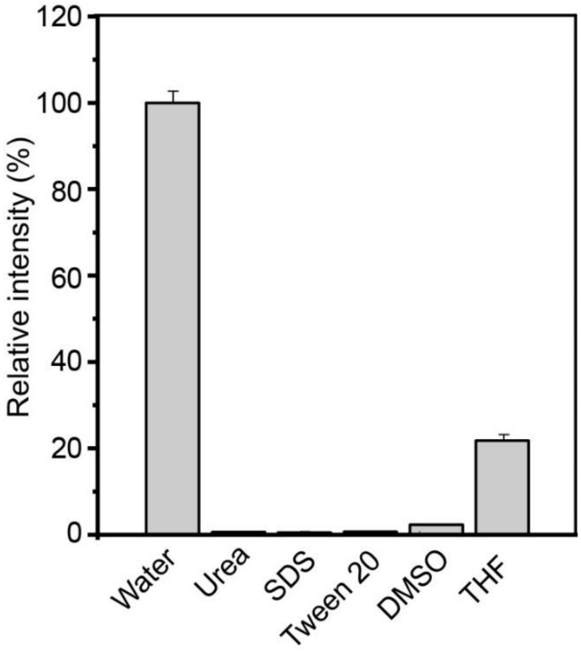

Figure S3. Stability of the yDNA–TA particles in various media, as measured by flow cytometry. The error bars represent standard deviations (n = 3).

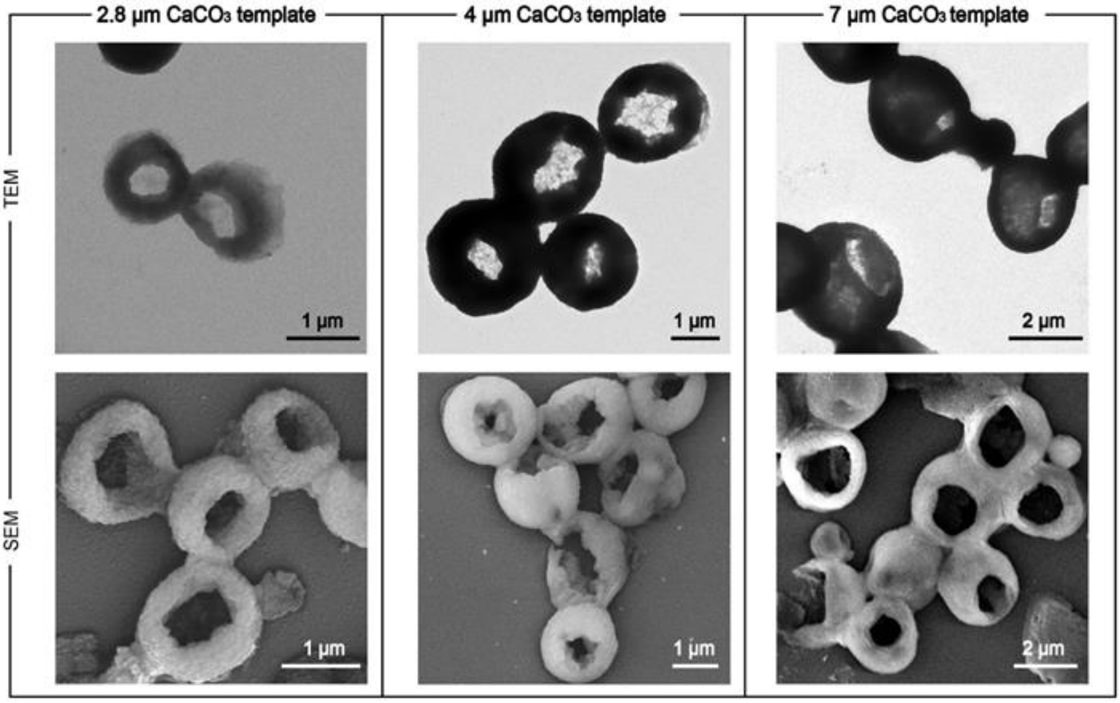

Figure S4. TEM and SEM images of the different yDNA–TA particles synthesized using $CaCO_3$ templates of different sizes.

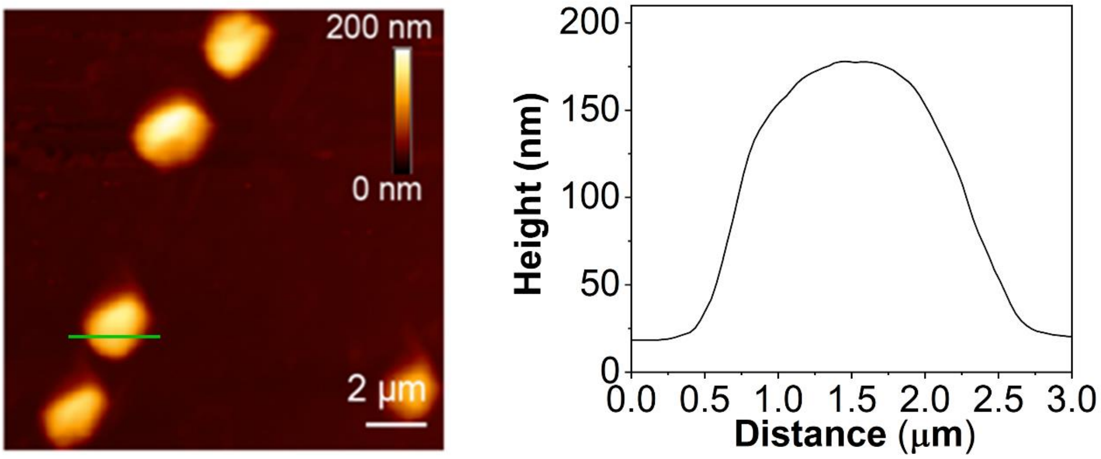

Figure S5. AFM image of the TA–yDNA capsules. The particles were prepared by reversing the deposition order of the building blocks (that is TA was deposited followed by yDNA). Height versus distance profile of a TA–yDNA capsule plotted along the green line in the AFM image.

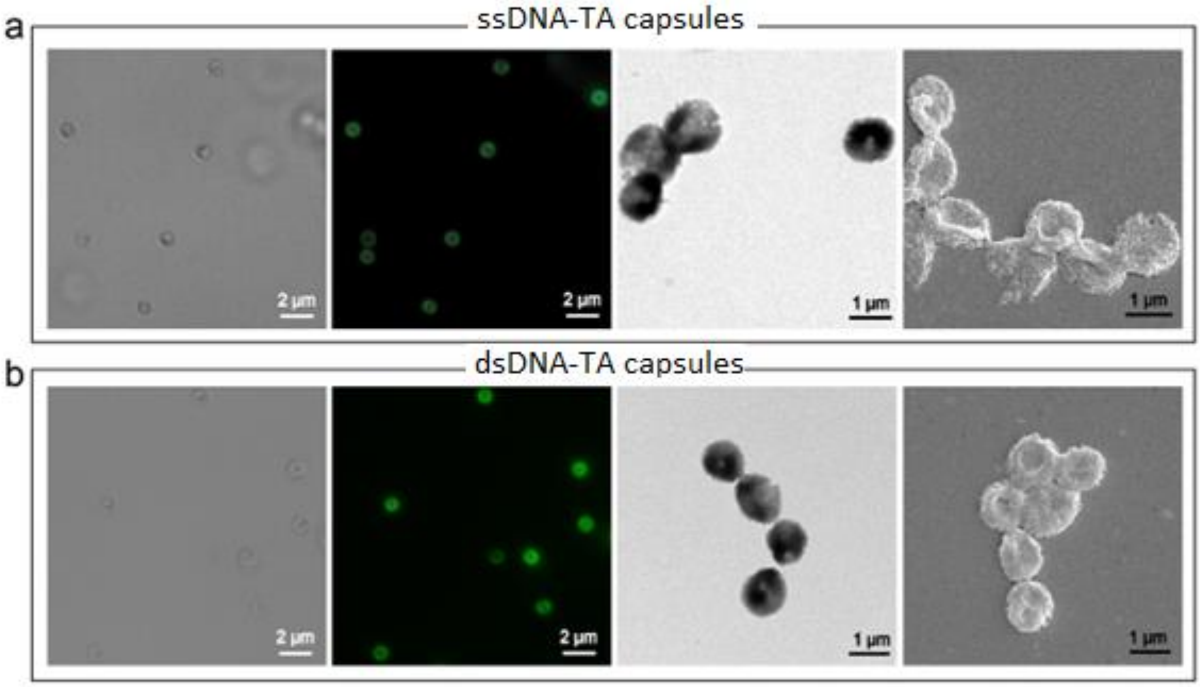

Figure S6. Differential interference contrast (DIC), CLSM, TEM, and SEM images of the (a) ssDNA–TA and (b) dsDNA–TA capsules.

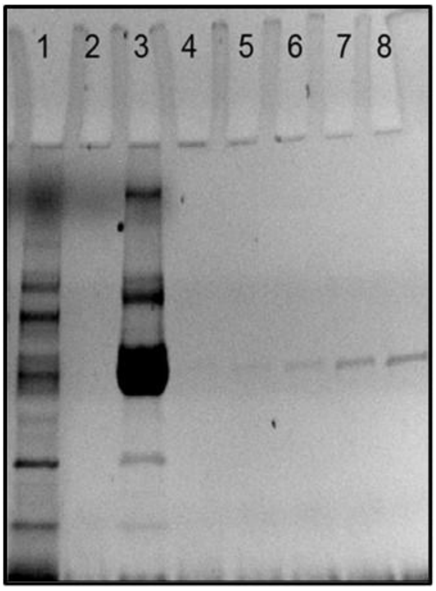

Figure S7. SDS-PAGE analysis of the proteins recovered from the yDNA–TA particles following incubation with DMEM containing 10% FBS. Proteins extracted from 2 × 10^8 yDNA–TA particles (lane 1); DMEM (lane 2); DPBS containing 10% FBS (lane 3); BSA standards: 50 ng (lane 4), 100 ng (lane 5), 200 ng (lane 6), 500 ng (lane 7), and 1 μg (lane 8).

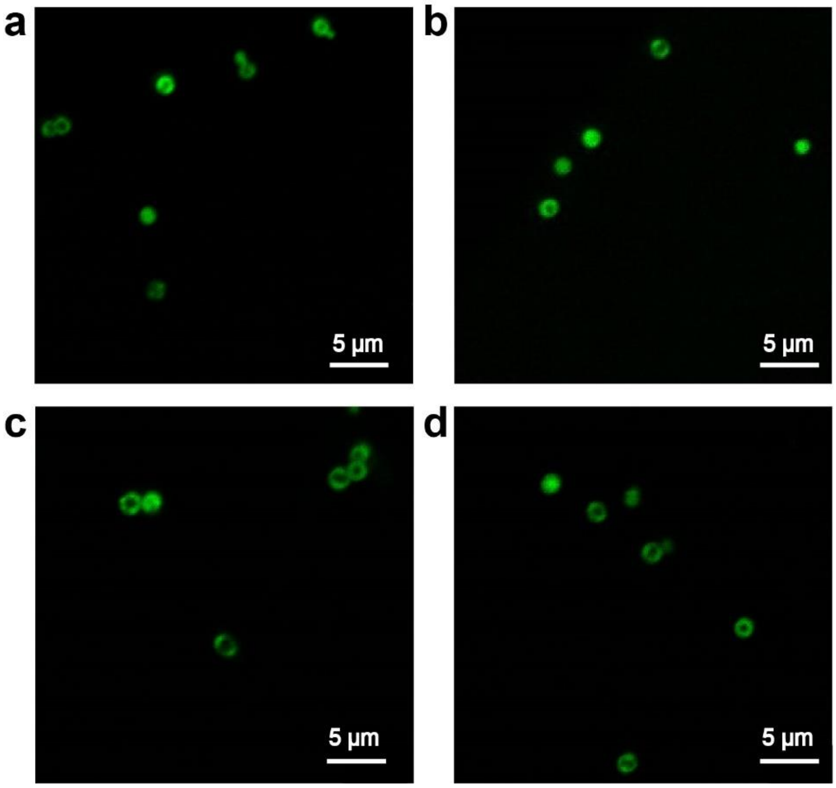

Figure S8. CLSM measurements of the stability of the yDNA–TA particles. The yDNA–TA particles were incubated in cell culture medium containing 10% FBS at 37 °C for (a) 2, (b) 4, (c) 8, and (d) 24 h prior to CLSM imaging.

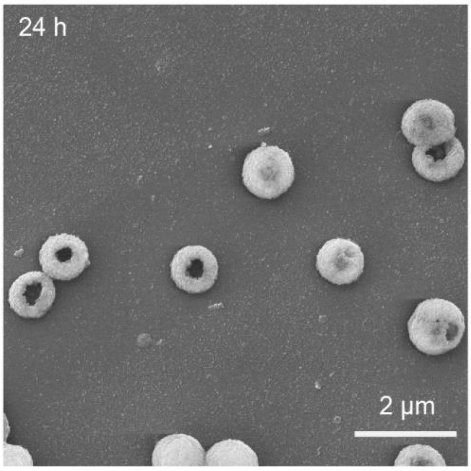

Figure S9. SEM image of the yDNA–TA particles incubated in 10% FBS at 37 °C for 24 h.

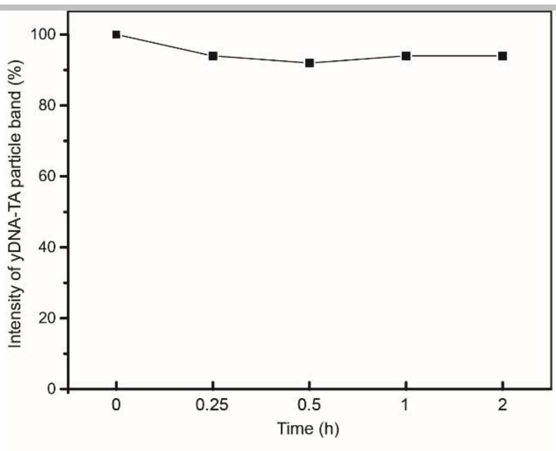

Figure S10. Intensity profile of the yDNA–TA particle band in agarose gel following incubation of the particles in 10% FBS medium at 37 °C.

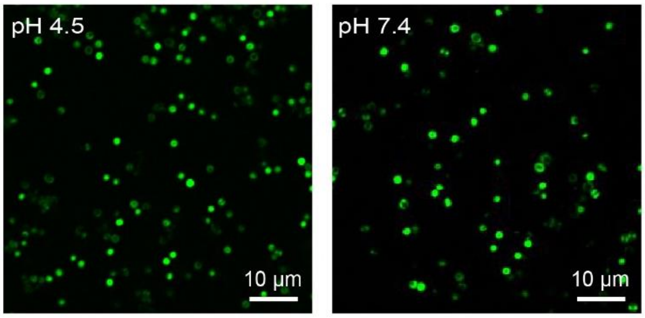

Figure S11. CLSM images of the yDNA–TA particles after incubation with different pH (4.5 and 7.4) cell culture media.

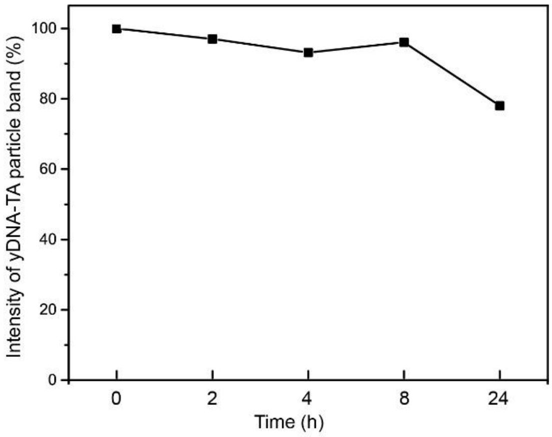

Figure S12. Intensity profile of the yDNA–TA particle band in agarose gel following incubation of the particles in DNase (1 U mL^{-1}) at 37 °C.

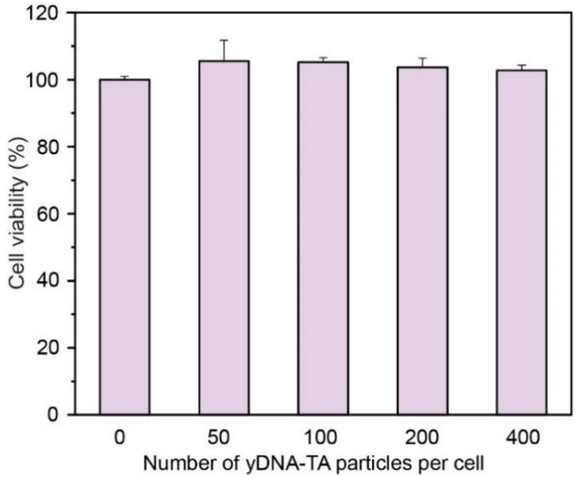

Figure S13. MTT assay of RAW264.7 cell viability measured after incubation of the yDNA–TA particles for 24 h at different particle-to-cell ratios.

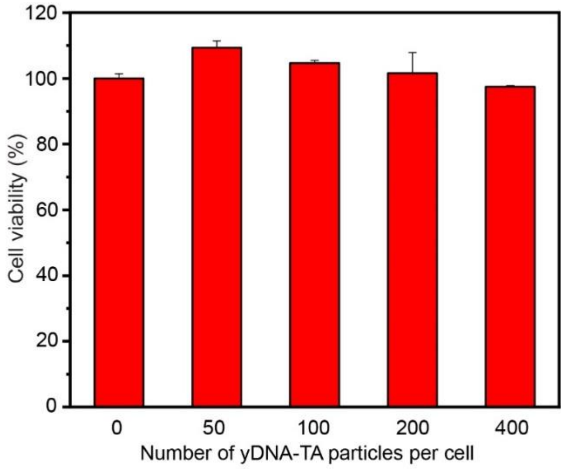

Figure S14. MTT assay of HeLa cell viability measured after incubation of the yDNA–TA particles for 24 h at different particle-to-cell ratios.

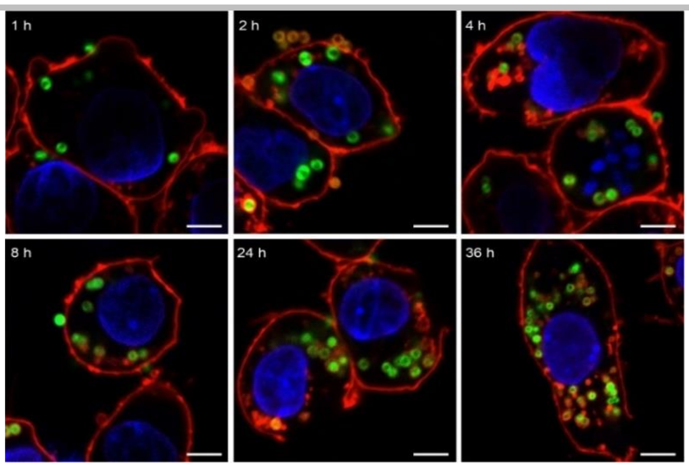

Figure S15. CLSM images of cells after incubation with the yDNA–TA particles for 1, 2, 4, 8, 24, and 36 h at a particle-to-cell ratio of 100:1. Green, AF488-labeled yDNA–TA particles; blue, nuclei; red, cell membrane. Scale bars: 5 μm.

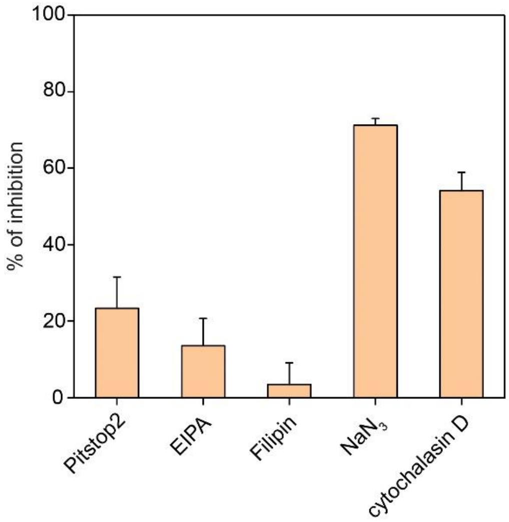

Figure S16. Study of the cell internalization mechanism of the yDNA–TA particles by monitoring the internalization efficiency in the presence of different endocytic inhibitors.

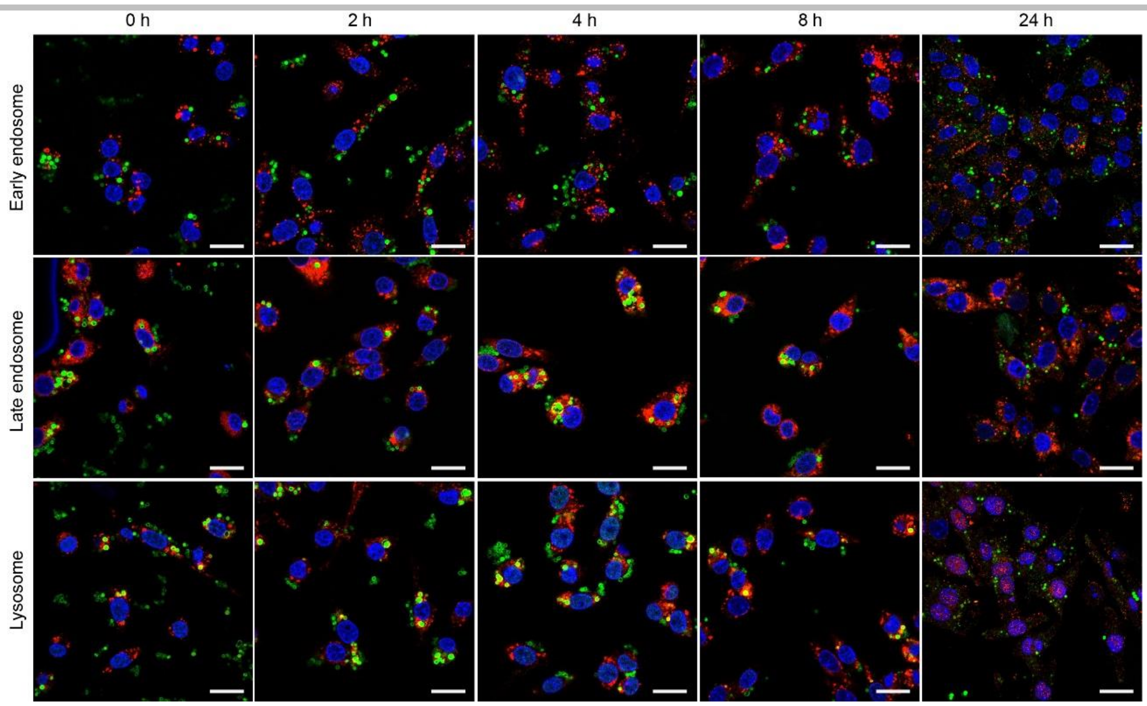

Figure S17. CLSM imaging of the intracellular trafficking of the yDNA–TA particles in RAW264.7 cells at varying incubation times of 0, 2, 4, 8, and 24 h (following initial incubation for 2 h). The particle-to-cell ratio was 100:1. Compartments in cells (red) were stained with EEA1 monoclonal antibody (early endosomes), anti-Rab7 monoclonal antibody (late endosomes), or anti-LAMP1 monoclonal antibody (lysosomes). Green, AF488-labeled yDNA–TA particles; blue, nuclei; red, early endosomes/late endosomes and lysosomes. Scale bars: 20 μm.

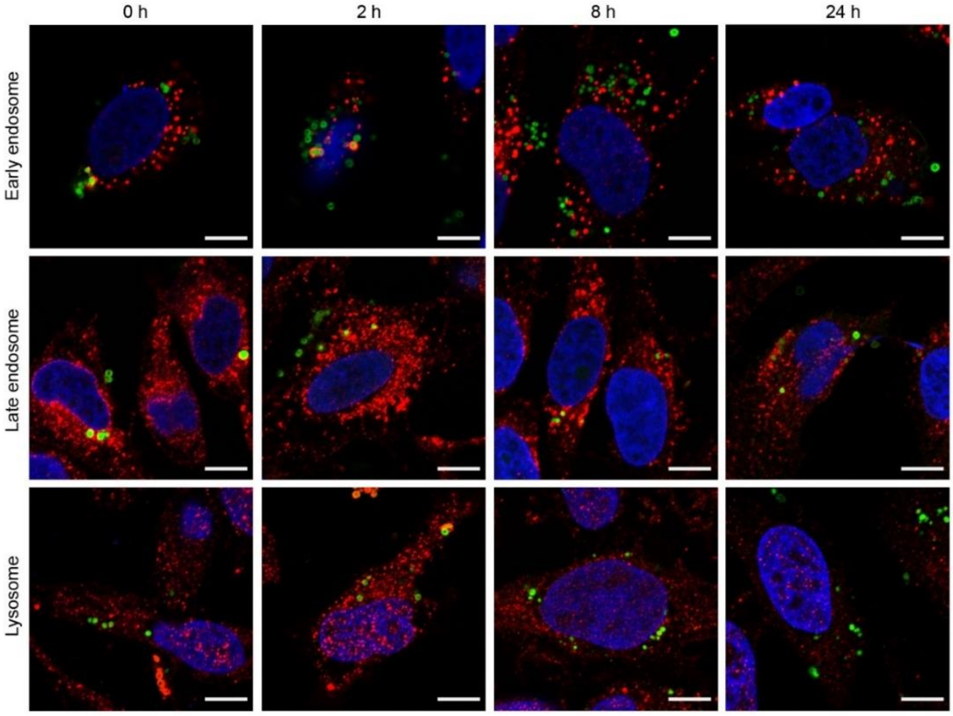

Figure S18. CLSM imaging of the intracellular trafficking of the yDNA–TA particles in HeLa cells at varying incubation times of 0, 2, 8, and 24 h (following initial incubation for 2 h). The particle-to-cell ratio was 100:1. Compartments in cells (red) were stained with EEA1 monoclonal antibody (early endosomes), anti-Rab7 monoclonal antibody (late endosomes), or anti-LAMP1 monoclonal antibody (lysosomes). Green, AF488-labeled yDNA–TA particles; blue, nuclei; red, early endosomes/late endosomes and lysosomes. Scale bars: 10 μm.

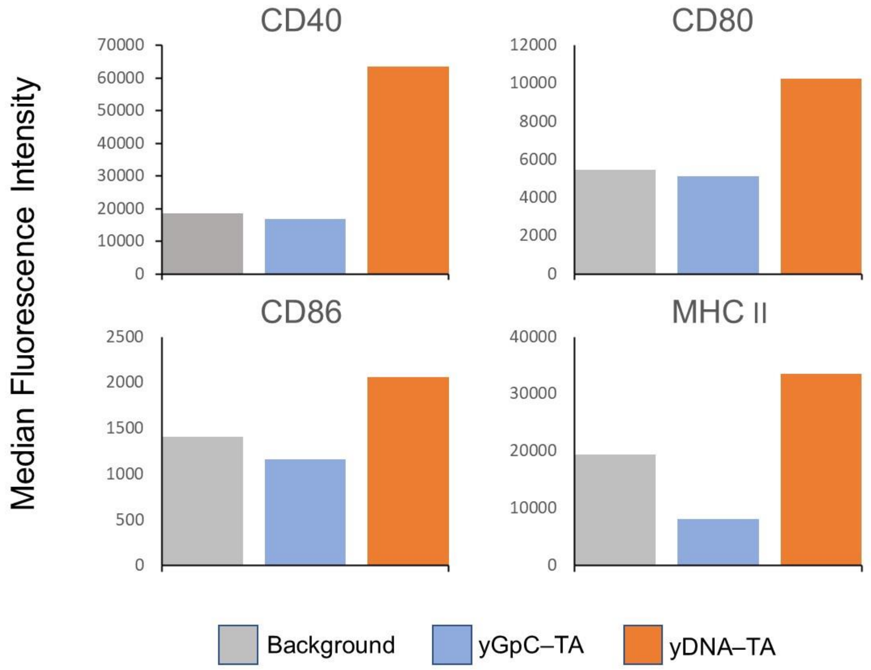

Figure S19. Activation of DC1940 dendritic cell line by CpG-containing microparticles, yDNA–TA. DC1940 cells were incubated with diluent (background), 500 nM of yGpC–TA (negative control) or yDNA–TA (stimulatory) particles at 37 °C/5% CO_2 for 15 h. Cells were labeled with antibodies to measure the expression of CD40, CD80, CD86, and MHC II receptors by flow cytometry (median fluorescence intensity).

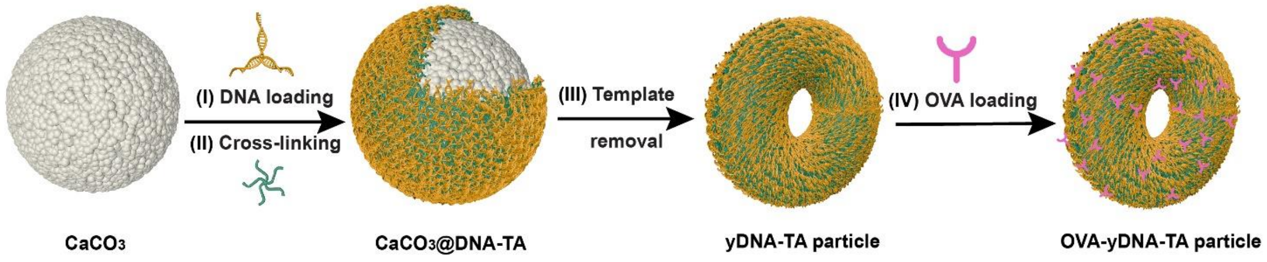

Figure S20. Schematic representation of the assembly of OVA–yDNA–TA particles: (I) loading of $CaCO_3$ particles with yDNA; (II) cross-linking by TA; (III) removal of $CaCO_3$ core template to yield yDNA–TA particles; and (IV) post-synthesis loading of OVA.

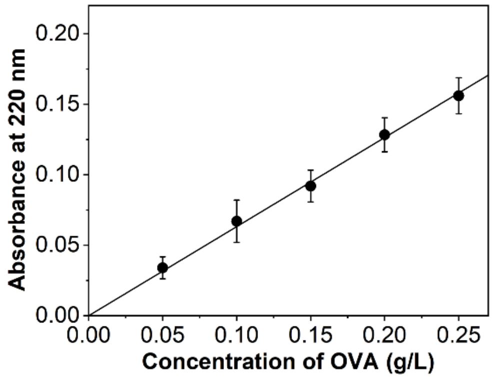

Figure S21. Standard curve of OVA.

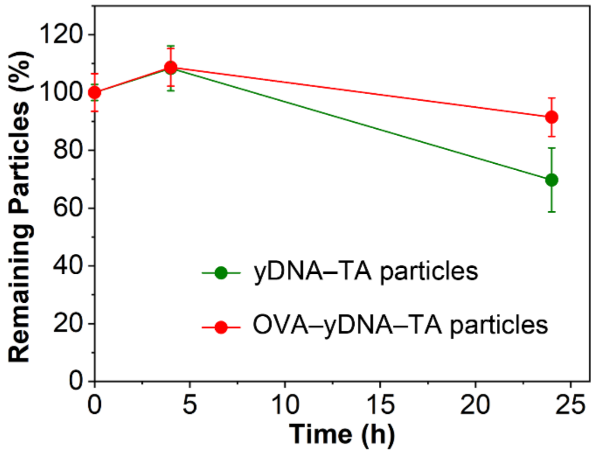

Figure S22. Comparison of the stability of yDNA–TA particles and OVA–yDNA–TA particles in serum, as assessed by flow cytometry. Data are shown as the mean ± standard deviation of three independent measurements.

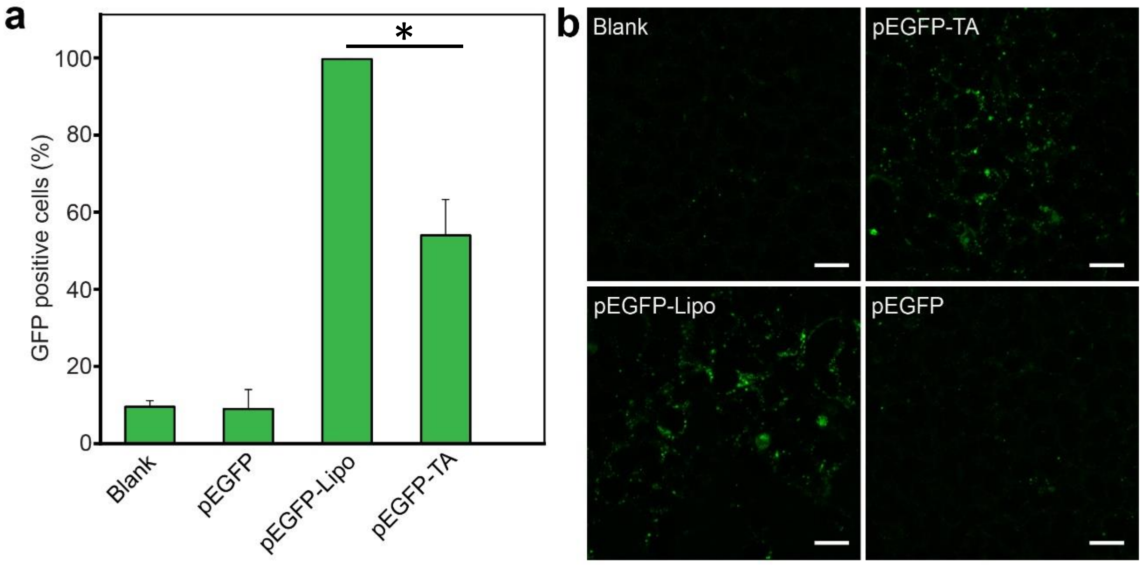

Figure S23. (a) In vitro transfection efficiency of pEGFP–TA measured in HEK293T cells using flow cytometry. Data are shown as the mean ± standard deviation (n = 3), *p < 0.05. (b) CLSM images of HEK293T cells after transfection with pEGFP–TA capsules (equivalent of 1 µg plasmid DNA per well and incubation for 72 h at 37 °C). pEGFP–lipofectamine (pEGFP–Lipo) complex and naked pEGFP were used as positive and negative controls, respectively. Scale bars: 20 µm.

Table S1. Sequence of oligonucleotides used in this work.

Strand	Sequence 2[a]
yDNA-1	TGCAAGCTGTTGAGTCGACGTTAAGAGCCTGCTGAAGCGTCTGCGA
yDNA-2	TGCAAGCTGTTGTCGCAGACGCTTCACTGAGCACAGACGTTGACGA
yDNA-3	TGCAAGCTGTTGTCGTCAACGTCTGTGCTCAGGCAGGCTCTTAACGTCGACT
ssDNA	TGCAAGCTGTTGTCGCAGACGCTTCACTGAGCACAGACGTTGACGA
dsDNA-1	CAA CAGCTT GCA TGC AGG CTT GAG CAG ACA GAG CCT TGA GCC TA
dsDNA-2	CAA CAG CTT GCA TAG GCT CAA GGC TCT GTC TGC TCA AGC CTG CA
yGpC-1	TGCAAGCTGTTGAGTGCAGCTTAAGAGCCTGCTGAAGGCTCTGGCA
yGpC-2	TGCAAGCTGTTGTGCCAGAGCCTTCACTGAGCACAGAGCTTGAGCA
yGpC-3	TGCAAGCTGTTGTGCTCAAGCTCTGTGCTCAGGCAGGCTCTTAAGCTGCACT

[a]The potent immunostimulatory CpG motif (GACGTT/GACGCT) is underlined.

Checklist

Minimum Information Reporting in Bio–Nano Experimental Literature

The MIRIBEL guidelines were introduced here: https://doi.org/10.1038/s41565-018-0246-4

The development of these guidelines was led by the ARC Centre of Excellence in Convergent Bio-Nano Science and Technology: https://www.cbns.org.au/. Any updates or revisions to this document will be made available here: http://doi.org/10.17605/OSF.IO/SMVTF. This document is made available under a CC-BY 4.0 license: https://creativecommons.org/licenses/by/4.0/.

The MIRIBEL guidelines were developed to facilitate reporting and dissemination of research in bio–nano science. Their development was inspired by various similar efforts:

- MIAME (microarray experiments): *Nat. Genet.* **29** (2001), 365; http://doi.org/10.1038/ng1201-365
- MIRIAM (biochemical models): *Nat. Biotechnol.* **23** (2005) 1509; http://doi.org/10.1038/nbt1156
- MIBBI (biology/biomedicine): *Nat. Biotechnol.* **26** (2008) 889; http://doi.org/10.1038/nbt.1411
- MIGS (genome sequencing): *Nat. Biotechnol.* **26** (2008) 541; http://doi.org/10.1038/nbt1360
- MIQE (quantitative PCR): *Clin. Chem.* **55** (2009) 611; http://doi.org/10.1373/clinchem.2008.112797
- ARRIVE (animal research): *PLOS Biol.* **8** (2010) e1000412; http://doi.org/10.1371/journal.pbio.1000412
- *Nature*'s reporting standards:
    - Life science: https://www.nature.com/authors/policies/reporting.pdf; e.g., *Nat. Nanotechnol.* **9** (2014) 949; http://doi.org/10.1038/nnano.2014.287
    - Solar cells: https://www.nature.com/authors/policies/solarchecklist.pdf; e.g., *Nat. Photonics* **9** (2015) 703; http://doi.org/10.1038/nphoton.2015.233
    - Lasers: https://www.nature.com/authors/policies/laserchecklist.pdf; e.g., *Nat. Photonics* **11** (2017) 139; http://doi.org/10.1038/nphoton.2017.28
- The "TOP guidelines": e.g., *Science* **352** (2016) 1147; http://doi.org/10.1126/science.aag2359

Similar to many of the efforts listed above, the parameters included in this checklist are **not** intended to be definitive requirements; instead they are intended as 'points to be considered', with authors themselves deciding which parameters are—and which are not—appropriate for their specific study.

This document is intended to be a living document, which we propose is revisited and amended annually by interested members of the community, who are encouraged to contact the authors of this document. Parts of this document were developed at the annual International Nanomedicine Conference in Sydney, Australia: http://www.oznanomed.org/, which will continue to act as a venue for their review and development, and interested members of the community are encouraged to attend.

After filling out the following pages, this checklist document can be attached as a "Supporting Information" document during submission of a manuscript to inform Editors and Reviewers (and eventually readers) that all points of MIRIBEL have been considered.

Supplementary Table 1. Material characterization*

**Question**	**Yes**	**No**
1.1 Are "**best reporting practices**" **available** for the nanomaterial used? For examples, see *Chem. Mater*. **28** (2016) 3535; http://doi.org/10.1021/acs.chemmater.6b01854 and *Chem. Mater*. **29** (2017) 1; http://doi.org/10.1021/acs.chemmater.6b05235		√
1.2 If they are available, **are they used**? If not available, ignore this question and proceed to the next one.		
1.3 Are extensive and clear instructions reported detailing all steps of **synthesis** and the resulting **composition** of the nanomaterial? For examples, see *Chem. Mater*. **26** (2014) 1765; http://doi.org/10.1021/cm500632c, and *Chem. Mater*. **26** (2014) 2211; http://doi.org/10.1021/cm5010449. Extensive use of photos, images, and videos are strongly encouraged. For example, see *Chem. Mater*. **28** (2016) 8441; http://doi.org/10.1021/acs.chemmater.6b04639	√	
1.4 Is the **size** (or **dimensions**, if non-spherical) and **shape of** the nanomaterial reported?	√	
1.5 Is the **size dispersity** or **aggregation** of the nanomaterial reported?	√	
1.6 Is the **zeta potential** of the nanomaterial reported?	√	
1.7 Is the **density (mass/volume)** of the nanomaterial reported?		√
1.8 Is the amount of any **drug loaded** reported? 'Drug' here broadly refers to functional cargos (e.g., proteins, small molecules, nucleic acids).	√	
1.9 Is the **targeting performance** of the nanomaterial reported, including **amount** of ligand bound to the nanomaterial if the material has been functionalised through addition of targeting ligands?	not applicable	
1.10 Is the **label signal** per nanomaterial/particle reported? For example, fluorescence signal per particle for fluorescently labelled nanomaterials.		√
1.11 If a material property not listed here is varied, has it been **quantified**?		√
1.12 Were characterizations performed in a **fluid mimicking biological conditions**?		√
1.13 Are details of how these parameters were **measured/estimated** provided?	√	
Explanation for **No** (if needed):		

***Ideally, material characterization should be performed in the same biological environment as that in which the study will be conducted. For example, for cell culture studies with nanoparticles, characterization steps would ideally be performed on nanoparticles dispersed in cell culture media. If this is not possible, then characteristics of the dispersant used (e.g., pH, ionic strength) should mimic as much as possible the biological environment being studied.

Supplementary Table 2. Biological characterization*

**Question**	**Yes**	**No**
2.1 Are **cell seeding details**, including **number of cells plated, confluency at start of experiment,** and **time between seeding and experiment** reported?	√	
2.2 If a standardised cell line is used, are the **designation and source** provided?	√	
2.3 Is the **passage number** (total number of times a cell culture has been subcultured) known and reported?	√	
2.4 Is the last instance of **verification of cell line** reported? If no verification has been performed, is the time passed and passage number since acquisition from trusted source (e.g., ATCC or ECACC) reported? For information, see *Science* **347** (2015) 938; http://doi.org/10.1126/science.347.6225.938		√
2.5 Are the results from **mycoplasma testing** of cell cultures reported?	√	
2.6 Is the **background signal of cells/tissue** reported? (E.g., the fluorescence signal of cells without particles in the case of a flow cytometry experiment.)	√	
2.7 Are **toxicity studies** provided to demonstrate that the material has the expected toxicity, and that the experimental protocol followed does not?	√	
2.8 Are details of media preparation (**type of media, serum,** any **added antibiotics**) provided?	√	
2.9 Is a **justification of the biological model** used provided? For examples for cancer models, see *Cancer Res.* **75** (2015) 4016; http://doi.org/10.1158/0008-5472.CAN-15-1558, and *Mol. Ther.* **20** (2012) 882; http://doi.org/10.1038/mt.2012.73, and *ACS Nano* **11** (2017) 9594; http://doi.org/10.1021/acsnano.7b04855	not applicable	
2.10 Is characterization of the **biological fluid** (*ex vivo*/*in vitro*) reported? For example, when investigating protein adsorption onto nanoparticles dispersed in blood serum, pertinent aspects of the blood serum should be characterised (e.g., protein concentrations and differences between donors used in study).	not applicable	
2.11 For **animal experiments**, are the ARRIVE guidelines followed? For details, see *PLOS Biol.* **8** (2010) e1000412; http://doi.org/10.1371/journal.pbio.1000412	not applicable	
Explanation for **No** (if needed):<br>2.4: Cells were purchased from the American Type Culture Collection. The passage number was reported and standard mycoplasma test was conducted.		

*For *in vitro* experiments (e.g., cell culture), *ex vivo* experiments (e.g., in blood samples), and *in vivo* experiments (e.g., animal models). The questions above that are appropriate depend on the type of experiment conducted.

Supplementary Table 3. Experimental details*

Question	Yes	No
3.1 For cell culture experiments: are **cell culture dimensions** including **type of well**, **volume of added media**, reported? Are cell types (i.e.; adherent vs suspension) and **orientation** (if non-standard) reported?	√	
3.2 Is the **dose of material administered** reported? This is typically provided in nanomaterial mass, volume, number, or surface area added. Is sufficient information reported so that regardless of which one is provided, the other dosage metrics can be calculated (i.e. using the dimensions and density of the nanomaterial)?	√	
3.3 For each type of imaging performed, are details of how **imaging** was performed provided, including details of **shielding, non-uniform image processing**, and any **contrast agents** added?	√	
3.4 Are details of how the dose was administered provided, including **method of administration, injection location, rate of administration,** and details of **multiple injections**?	Not applicable	
3.5 Is the methodology used to **equalise dosage** provided?	√	
3.6 Is the **delivered dose** to tissues and/or organs (in vivo) reported, as % injected dose per gram of tissue (%ID g^{-1})?	Not applicable	
3.7 Is **mass of each organ/tissue measured** and **mass of material** reported?	Not applicable	
3.8 Are the **signals of cells/tissues with nanomaterials** reported? For instance, for fluorescently labelled nanoparticles, the total number of particles per cell or the fluorescence intensity of particles + cells, at each assessed timepoint.	√	
3.9 Are **data analysis details**, including **code used** for analysis provided?	√	
3.10 Is the **raw data** or **distribution of values** underlying the reported results provided? For examples, see *R. Soc. Open Sci.* **3** (2016) 150547; http://doi.org/10.1098/rsos.150547, https://opennessinitiative.org/making-your-data-public/, http://journals.plos.org/plosone/s/data-availability, and https://www.nature.com/sdata/policies/repositories		√
Explanation for **No** (if needed):		

* The use of protocol repositories (e.g., *Protocol Exchange* http://www.nature.com/protocolexchange/) and published standard methods and protocols (e.g., *Chem. Mater.* **29** (2017) 1; http://doi.org/10.1021/acs.chemmater.6b05235, and *Chem. Mater.* **29** (2017) 475; http://doi.org/10.1021/acs.chemmater.6b05481) are encouraged.

References

[1] Y. Qu, Y. Ju, C. Cortez-Jugo, Z. Lin, S. Li, J. Zhou, Y. Ma, A. Glab, S. J. Kent, F. Cavalieri, F. Caruso, *Small* **2020**, *16*, 2002750.

[2] M. Faria; M. Björnmalm; K. J. Thurecht; S. J. Kent; R. G. Parton; M. Kavallaris; A. P. Johnston; J. J. Gooding; S. R. Corrie; B. J. Boyd; P. Thordarson; A. K. Whittaker; M. M. Stevens; C. A. Prestidge; C. J. H. Porter; W. J. Parak; T. P. Davis; E. J. Crampin; F. Caruso *Nat. Nanotechnol*. **2018**, *13*, 777–785.

Author Contributions

Y.Q .initiated the project, conducted the in vitro experiments and data analysis, and wrote the original draft of the manuscript; R.D.R. designed and conducted the in vivo experiments and wrote the original draft of the manuscript. C.-J.K. optimized the production of materials for in vivo studies. J.Z. assisted with experimental design, experiment troubleshooting and TEM imaging; Z.L. assisted with discussions for troubleshooting, TEM imaging, SEM imaging and scheme design; Y.J. assisted with flow cytometry data analysis and discussion; S.K.B. assisted with CLSM and discussion; C.C.-J assisted with discussion; F. Cavalieri and F. Caruso provided guidance on scientific experiments and data analysis. All authors contributed to the revision of the manuscript at varying draft stages.
